# Factors associated with the prevalence of HIV, HSV-2, pregnancy, and reported sexual activity among adolescent girls in rural western Kenya: A cross-sectional analysis of baseline data in a cluster randomized controlled trial

**DOI:** 10.1371/journal.pmed.1003756

**Published:** 2021-09-28

**Authors:** Garazi Zulaika, Elizabeth Nyothach, Anna Maria van Eijk, David Obor, Linda Mason, Duolao Wang, Tao Chen, Emily Kerubo, Valarie Opollo, Isaac Ngere, Samuel Omondi Owino, Boaz Oyaro, Feiko O. ter Kuile, Daniel Kwaro, Penelope Phillips-Howard

**Affiliations:** 1 Liverpool School of Tropical Medicine (LSTM), Liverpool, United Kingdom; 2 Kenya Medical Research Institute (KEMRI), Centre for Global Health Research, Kisumu, Kenya; 3 Ministry of Health, Siaya County, Kenya; Emory University, UNITED STATES

## Abstract

**Background:**

Adolescence is a sensitive time for girls’ sexual and reproductive health (SRH), as biological changes occur concurrently with heightening pressures for sexual activity. In western Kenya, adolescent girls are vulnerable to acquiring sexually transmitted infections (STIs), such as HIV and herpes simplex virus type 2 (HSV-2), and to becoming pregnant prior to reaching adulthood. This study examines associations between individual, household, and partner-related risk factors and the prevalence of sex, adolescent pregnancy, HIV, and HSV-2.

**Methods and findings:**

We report baseline findings among 4,138 girls attending secondary school who were enrolled between 2017 and 2018 in the Cups or Cash for Girls (CCG) cluster randomized controlled trial in Siaya County, rural western Kenya. Laboratory confirmed biomarkers and survey data were utilized to assess the effects of girls’ individual, household, and partner characteristics on the main outcome measures (adolescent reported sex, prior pregnancy, HIV, and HSV-2) through generalized linear model (GLM) analysis. Complete data were available for 3,998 girls (97%) with median age 17.1 years (interquartile range [IQR] 16.3 to 18.0 years); 17.2% were HSV-2 seropositive (*n =* 686) and 1.7% tested positive for HIV (*n =* 66). Sexual activity was reported by 27.3% girls (*n =* 1,090), of whom 12.2% had been pregnant (*n* = 133). After adjustment, orphanhood (adjusted risk ratio [aRR] 2.81, 95% confidence interval [CI] 1.18 to 6.71, *p*-value [p] = 0.020), low body mass index (BMI) (aRR 2.07; CI: 1.00 to 4.30, *p* = 0.051), and age (aRR 1.34, 1.18 to 1.53, *p* < 0.001) were all associated with HIV infection. Girls reporting light menstrual bleeding (aRR 2.42, 1.22 to 4.79, *p* = 0.012) for fewer than 3 days (aRR 2.81, 1.16 to 6.82, *p* = 0.023) were over twice as likely to have HIV. Early menarche (aRR 2.05, 1.33 to 3.17, *p* = 0.001) was associated with adolescent pregnancy and HSV-2–seropositive girls reported higher rates of pregnancy (aRR 1.62, CI: 1.16 to 2.27, *p* = 0.005). High BMI was associated with HSV-2 (aRR 1.24, 1.05 to 1.46, *p* = 0.010) and sexual activity (aRR 1.14, 1.02 to 1.28, *p* = 0.016). High levels of harassment were detected in the cohort (41.2%); being touched indecently conveyed the strongest association related to reported sexual activity (aRR 2.52, 2.26 to 2.81, *p* < 0.001). Study limitations include the cross-sectional design of the study, which informs on the SRH burdens found in this population but limits causal interpretation of associations, and the self-reported exposure ascertainment, which may have led to possible underreporting of risk factors, most notably prior sexual activity.

**Conclusions:**

Our findings indicate that adolescent girls attending school in Kenya face frequent harassment for sex and are at high risk of pregnancy and HSV-2, with girls experiencing early menarche particularly vulnerable. Targeted interventions, such as earlier sexual education programs, are warranted to address their vulnerability to SRH harms.

**Trial registration:**

ClinicalTrials.gov NCT03051789.

## Abstract 2

In a cross sectional analysis, Garazi Zulaika and colleagues study associations between risk factors and prevalence of sexual activity, pregnancy, and HIV and HSV-2 infections among adolescent girls in rural Kenya.

## Author summary

**Why was this study done?:**

- Girls in low- and middle-income countries (LMICs) face severe health risks from becoming pregnant prior to reaching adulthood and from exposure to sexually transmitted infections (STIs) such as HIV and herpes simplex virus type 2 (HSV-2).
- Key factors found to be linked to adolescent sexual and reproductive health (SRH) in LMICs include household wealth, age at first sex, and schooling.
- Evidence of a positive association between girls’ SRH, and their education and economic potential has strengthened international resolve to identify risks and improve conditions that adolescent girls face in these settings.

**What did the researchers do and find?:**

- We collected data from 3,998 girls attending 96 public secondary day schools in 2017 and 2018 as part of the baseline assessment of a randomized controlled study.
- We explored whether girls’ personal characteristics were associated with SRH harms, namely adolescent sex, early pregnancy, and acquisition of HIV and HSV-2.
- We found biological factors including girls experiencing early menarche, having lighter periods, and body mass index (BMI); household level factors including being unhappy at home, being orphaned, and coming from poorer households; and social factors including experiencing harassment for sex both in and out of school or being touched indecently were significantly associated with girls’ sexual and reproductive harms, such as pregnancy, HSV-2, and/or HIV.

**What do these findings mean?:**

- These findings illustrate that the burden of SRH harms faced by adolescent girls attending school in rural western Kenya is high.
- Interventions and programs to improve SRH and continued education need to address both environmental and biological risk factors among vulnerable populations.

## Introduction

Adolescence is a sensitive time for female sexual and reproductive health (SRH), as biological changes occur concurrently with heightening social and sexual pressures [1,2]. Girls’ biological immaturity and social vulnerability leave them with limited awareness or agency to navigate relationships and sexual encounters [3]. Girls may also be exposed to sexual abuse or live in environments with high rates of gender inequality or where economic opportunities for girls are scarce [4]. These factors, in turn, make girls disproportionately susceptible to acquiring sexually transmitted infections (STIs), such as HIV and herpes simplex virus type 2 (HSV-2), or to becoming pregnant [3,5–8].

In Africa, 26% of deaths in females aged 10 to 24 years are due to maternal causes, with HIV-related deaths closely trailing [9,10]. Worldwide, 11% of births occur in girls aged 15 to 19, with approximately 95% of these occurring in low- and middle-income countries (LMICs) [11]. It has been widely evidenced that pregnancy at a young maternal age poses severe health risks for mother and baby, with infant deaths 50% higher among those born to adolescent mothers than women in their 20s, and heightened maternal mortality due to disproportionate rates of pregnancy complications and induced abortions [12,13]. More recently, in Kenya, evidence is building showing heightened levels of depression in adolescent mothers due to social stigma and isolation, lack of financial and emotional support, and poor access to health services [11]. These short- and long-term physical and mental health harms are often accompanied by interruptions in education, skill development, and the formation of social networks [14,15]. This reality underscores the importance of girls’ reproductive health and the central role that risky sexual behaviors (i.e., age at sexual debut, number of sexual partners, and condom use) have on adolescent girls’ life and economic prospects.

In much of sub-Saharan Africa, including Kenya, adolescent girls and women are at the epicenter of the HIV epidemic [16,17]. In Kenya, new HIV infections among adolescent girls aged 15 to 24 years were more than double that of boys (11,000 versus 5,000 in 2018) [18]. HSV-2 and HIV prevalence have been shown to be strongly associated, with evidence suggesting that HSV-2 can be used as a “temperature scale” to measure the intensity of sexual risk behaviors that drive HIV transmission, and to identify high-risk populations [19]. HSV-2 is the most common cause of genital ulcer disease worldwide, and the most prevalent STI in sub-Saharan Africa, making it a well-established biomarker for sexual risk [20–22]. Moreover, many studies have shown that HSV-2 seropositivity increases the risk of HIV acquisition by as much as 3-fold and may also increase HIV transmission [23].

Girls in western Kenya have the youngest age of sexual debut, first marriage, and first birth in the country [24]. SRH harms are reportedly high, with one study showing 45% of sexually active girls being coerced into first sex [25] and another reporting 52% of sexually active girls engaging in transactional sex for money, gifts, or services [26]. While, nationally, 25% of girls become pregnant during adolescence, in western Kenya, estimates indicate over 60% of girls enter motherhood by the time they reach age 19 [24]. The area also sustains high HSV-2 prevalence, with a quarter of incident HIV infections possibly attributable to HSV-2 infection [27]. The 2018 National Kenya AIDS Indicator Survey showed that Siaya County, western Kenya had the highest prevalence of HIV, at 21% among 15- to 64-year-olds despite wide provision of HIV diagnostic and care services [28]. Among adolescents, nearly 17,000 youths aged 15 to 24 years live with HIV in Siaya County, and the region contributed over 9% of new infections among adolescents in Kenya in 2017 [28]. For HSV-2, a steep increase in prevalence is seen by age, with figures ranging from 10% in 13- to 14-year-olds, 28% in 15- to 19-year-olds, to 70% among the 20- to 24-year-olds [29,30].

This study utilizes baseline data from the Cups or Cash for Girls (CCG) cluster randomized controlled trial in 5 subcounties across western Kenya among girls enrolled in secondary school. It examines the associations between key adolescent sociodemographic and behavioral correlates and the risk of sexual activity, adolescent pregnancy, HIV, and HSV-2.

## Methods

### Study design

This study presents cross-sectional baseline data collected at enrollment for the CCG Trial evaluating the effect of conditional cash transfer and/or menstrual cups on a composite of deleterious outcomes, described in detail elsewhere [31]; ClinicalTrials.gov NCT03051789.

### Study area and population

The study took place in 96 secondary day schools in Siaya County, western Kenya. The area borders Lake Victoria to the south and Kisumu City 40 km to the east. Study schools were rural or peri-urban and spread across approximately 2,500 km^2^ in the Gem, Siaya, Rarieda, Ugenya, and Ugunja subcounties. Siaya is a relatively poor area with a health profile that typifies much of rural Africa, with high endemicity of malaria, HIV, TB, and schistosomiasis close to the lake shores [32]. The population are predominantly ethnic Luo who are traditionally farmers and fisherfolk [33].

In 2015, 24% of the female population around Lake Victoria were girls aged 10 to 19 years [34]. Adolescent girls in this area have a heightened risk profile for poor SRH outcomes. The median age of first sex has remained low at 16.6 years, and early pregnancy is common [35,36]. The region has the lowest median age at first birth in the country at 18.9 years [35]. Studies in the area have measured maternal mortality ratios of 669 per 100,000 live births, with a 1 in 26 lifetime risk of dying [37]; these risks are disproportionately weighted toward biologically underdeveloped younger girls. Additionally, HIV rates among adolescent girls are nearly twice that of boys in this area, with girls reporting high rates of domestic violence and sexual coercion [17,38].

The CCG Trial targeted female students attending secondary day schools in the study area [31]. Study schools were eligible if they schooled female day scholars (nonboarders) and had head teacher’s (school principal’s) approval to participate. Schools were excluded if they were special needs schools (i.e., schools for the blind), boys only, or full boarding. Participants were eligible if they were female, resident of the area, attending an eligible secondary day school, were day scholars in the designated class years at enrollment, had informed parent–guardian consent and gave their individual informed assent to participate, had reached menarche, were not visibly or declared pregnant at the time of enrollment, and had no disability precluding participation [31].

### Study recruitment and procedures

Meetings were held with the Ministry of Education at the national and county levels to acquire approvals and permissions to work within the schools. Meetings were also conducted with the Ministry of Health at the subcounty, county, and national levels to consider the health-related aspects of the trial. Study-related activities were discussed with all school principals, village chiefs, and clinic staff to obtain feedback prior to launching the study. A census of area secondary schools was used to identify eligible schools. Sample size and power calculations were performed for the minimum number of schools and female students needed for the primary comparisons in the proposed 4-arm trial using NCSS/PASS sample size software; calculations were validated using SAS-based simulation studies. A total of 96 school clusters with an anticipated average of 41.25 girls per cluster were needed to allow for the primary trial comparisons, yielding a full sample of 3,960 girls overall [31]. The trial statistician block-randomized groupings of 4 schools using a 1:1:1:1 ratio based on school size within each subcounty; the largest 24 school blocks were recruited to obtain an adequate sample. Principals representing eligible schools were invited to participate in public randomization ceremonies in which they blindly selected 1 of 4 coded items within their blocks to be allocated an intervention arm [31]. School enrollment registers of participating schools were used to identify the participant sampling frame.

**Fig 1:**
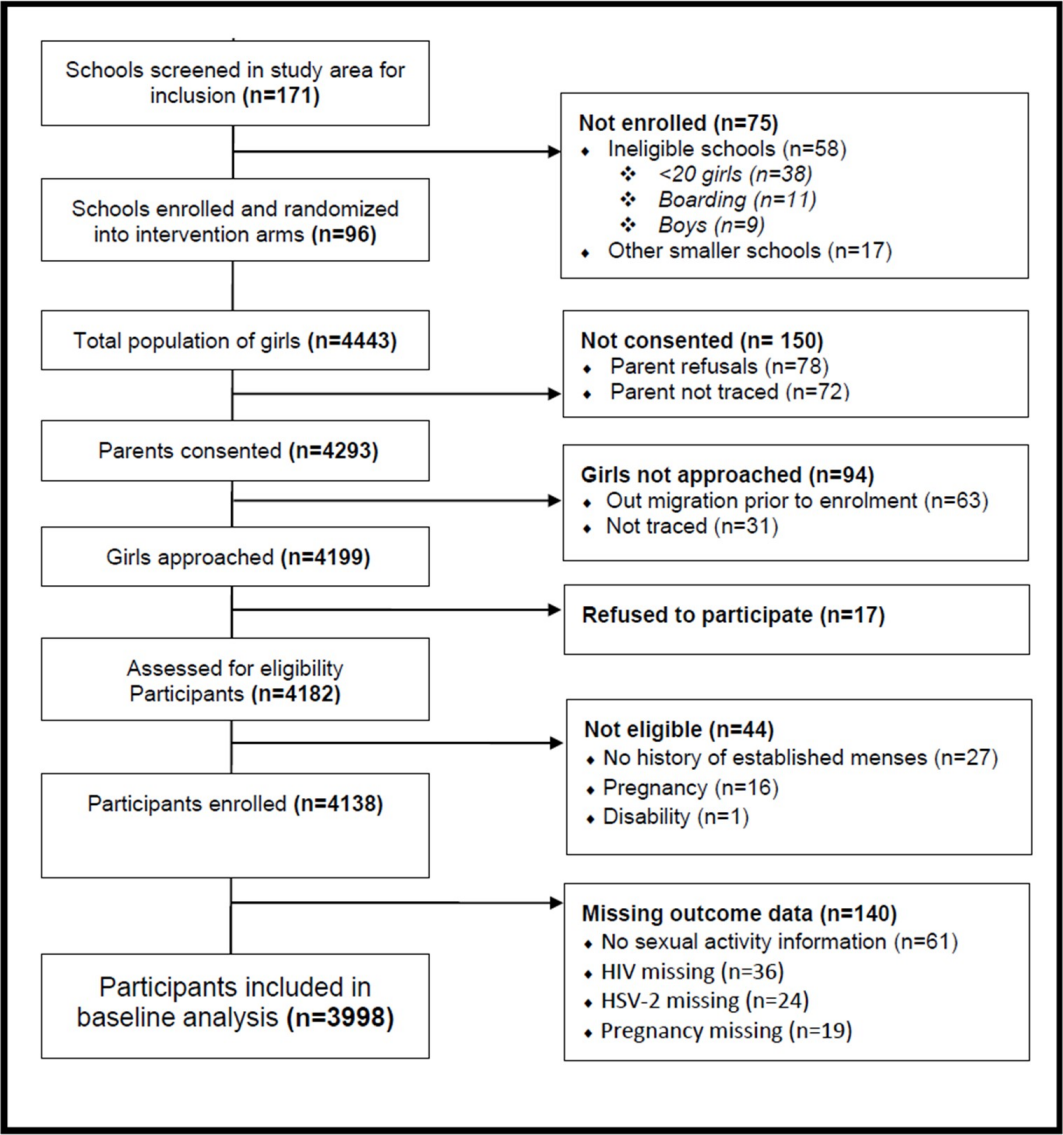
Flow diagram of CCG Trial participant inclusion.

Girls’ parent–guardians were invited to a school-based meeting to discuss the study objectives and timelines. Parent–guardians were invited to provide their written informed consent for participants under the age of 18 to allow their daughter or ward to participate. Informed consent for blood specimen collection and HIV and HSV-2 testing was obtained separately. Once a parent–guardian provided consent, school meetings were scheduled with eligible girls to inform them of the study and explain study procedures. Girls who were willing to participate were asked to provide written informed assent prior to being enrolled in the study [31]. Only girls with complete outcome data were included in this analysis (Fig 1).

Once informed consent and assent was obtained, girls were screened for eligibility by a study counselor and enrolled into the study. Enrolled participants were familiarized with Android-based tablets and invited to complete a self-administered sociodemographic and behavioral survey (S1 Survey) using forms built on Open Data Kit (ODK). Dual English and Luo translations were used. Behavioral survey questions including details on adolescent cash use were adopted from previously vetted tools in the same area [39,40]. Questions to measure socioeconomic status (SES) were adopted from the KEMRI/CDC Health and Demographic Surveillance System [41,42], gender-based violence from the Kenya Demographic and Health Surveys [35], and adolescent well-being from prevalidated PedsQL-23 instruments [43]. Participants individually received HIV counseling prior to providing a blood specimen for HIV and HSV-2 testing. While samples were collected at school, HIV and HSV-2 results were returned to participants’ selected health facilities to maintain confidentiality and provide direct linkage to care. Additionally, participant height, weight, hip, and waist anthropometric measurements were captured [31].

### Laboratory procedures

Samples were transported in microtainer EDTA tubes to KEMRI laboratories for analysis. HIV testing at baseline was conducted sequentially using Determine, confirmed with Unigold, with the third-generation Bio Rad ELISA as the tie-breaker [44]. HSV-2 was examined using the Kalon IgG2 ELISA test kit (Kalon Biologicals, Guilford, United Kingdom) [45].

### Data processing and analysis

Of the 4,138 participants enrolled, 3,998 girls (96.6%) with data on all outcomes of interest (HIV, HSV-2, and sexual activity) were included in this complete case analysis. Data captured on tablets were deidentified at the source and linked with laboratory results for HIV and HSV-2 and anthropometry. All indeterminate HSV-2 laboratory results (3.4%) were conservatively classified as negative [46], which may underestimate the true HSV-2 positivity rate in the population.

Using generalized linear models (GLMs), we estimated the risk ratios (RRs) and 95% confidence intervals (95% CIs) of key covariates and risk factors against prevalent HIV and HSV-2 infections, history of pregnancy, and reported sexual activity at baseline. A sexual activity response variable was constructed, comprising of either those directly reporting having had sexual intercourse and/or those reporting being tricked or forced into having sexual intercourse. History of pregnancy was self-reported in the baseline survey.

Covariates of interest were selected due to (1) their established importance in the existing literature; (2) being locally relevant in our study population and setting; and (3) key to understanding girls’ vulnerability to study outcomes. All analyses presented here were planned prior to survey development, although no prospective analysis plan was developed for this descriptive baseline analysis. Covariates explored included individual characteristics (age, body mass index [BMI], and sexual activity), family characteristics (marital status, caring for a baby at home, and having no living parent), household characteristics (SES), lifestyle characteristics (self-reported alcohol use, smoking, and working outside of school and home), well-being (harassment at school, harassment out of school, being happy at home, being happy at school, and quality of life score for well-being [PedsQL-23]), menstruation-related characteristics (year of menarche, early menarche, using sanitary pads for menstruation, menstruation-related absence, stopping daily activities due to menstruation, severity of menstruation, duration of bleeding, and having to do things like housework, childcare, or sex work to obtain menstrual pads), and financial characteristics (source of money and transactional sex). For girls who were sexually active, separate models were built for HSV-2 and pregnancy, with covariates including individual-level factors (age at sexual debut, sexual intent, number of lifetime partners, use of condoms, use of family planning, and transactional sex) and partner characteristics (age of partner, whether partner was someone girl knew, relationship to partner, and partner circumcision status).

Certain covariates were dichotomized into 2 response profiles: (1) early menarche was defined as reaching menarche prior to age 13 [47]; (2) early sex was defined as sexual debut prior to age 15; (3) marital status was grouped as “married, cohabiting, or widowed” versus “single, other”; (4) having no living parent “no living parent” versus “one or both parents alive”; and (5) who did you have sex with for the first time “a partner, boyfriend, husband” or “someone else.” Severity of menstrual bleeding was self-reported as “heavy,” “normal,” or “light.” BMI was calculated from participants’ anthropometric measurements. Percentile ranks were used as a criterion to classify adolescents in to predetermined body weight categories [48]. Girls with BMIs in the bottom fifth percentile were classified as “underweight” (BMI <18.2), “normal weight” ranged between BMI 18.2 to 25, and “overweight” comprised BMI >25 (S1 Fig).

To measure SES, we constructed an absolute index (S2 Fig) based on girls’ reported household assets following the methodology outlined in Kabudula and colleagues [49]. Once an SES value was assigned to each participant, they were split into 5 quintiles and subgrouped as “poorest” (quintiles 1 to 2) and “less poor” (quintiles 3 to 5). To construct the predictor variable for girls’ overall well-being, the PedsQL-23 tool was used. The PedsQL uses 23 individual items to group girls’ well-being into 4 categories: physical, emotional, social, and school well-being [43]. For each of the 4 dimensions, Likert scaled item answers were reverse scored and linearly transformed. Items were grouped by category, and mean scores for each category were computed. A low well-being score was classified as a numeric value between 0 to 25, moderately low for a score of 26 to 50, moderate for a score of 51 to 75, and high well-being for a score of 76 to 100 [43]. Overall well-being was computed by taking the mean of all 23 individual items, with higher mean scores indicating better well-being. Lastly, the remaining covariates were left as multiresponse category variables or continuous variables (i.e., age).

Descriptive statistics were used to summarize participant characteristics across the full sample, and separately for sexually active girls, HIV and HSV-2–positive girls, and for those with a history of pregnancy. Unadjusted univariate and adjusted multivariable GLMs were constructed to estimate risk ratios (RRs, adjusted risk ratios [aRRs], 95% CIs) for the associations between key predictors and each of the 4 binary response variables in STATA SA 14.0 (StataCorp LP, College Station, Texas, United States of America) in the full sample and separately among girls reporting sexual activity. The models were fit using a binomial distributions and a log link function with robust SE adjustment for clustering by school; Poisson distributions were used to assess risk in the case of failed model convergence [50]. We tested variance inflation factor (VIF) values in models where collinearity could be suspected. If an explanatory variable was highly collinear with an outcome of interest (e.g., having a child at home to care for and having previously been pregnant), the explanatory variable was dropped from the model. If 2 explanatory variables were collinear (VIF > 2.0), then they were investigated separately and the variable with most clinical relevance was retained. Normality was checked through histograms; linearity of association of continuous covariates was assessed graphically through the STATA lincheck GLM option. For associations that were nonlinear, covariates were treated as categorical variables (i.e., mean overall well-being score). Covariates found to be significant in the univariate analysis were added to the overall multivariable model through stepwise regression procedures for model fit and retained in the final model at *p* < 0.05. The STATA GLM bootstrapping option was employed to quality check the multivariable models. Models with the full exposure–outcome relationships were bootstrapped at 1,000 replications; all covariates significant at *p* < 0.2 were entered into a multivariable model and checked against the results of the stepwise regression. If the variable selection selected factors not already in the stepwise mode, model fit was assessed and potential confounders were added to the model even if they were not significant in the univariate analysis. Lastly, the STATA swboot procedure was performed at 1,000 replications to cross-validate the variable selection in the main stepwise models (S1 Table).

### Ethics approval and consent to participate

All participation was voluntary; girls could withdraw from the study at any time. The protocol, written informed parent–guardian consent and participant assent documents, and information sheets were reviewed and approved by the Research Ethics Committees at KEMRI, Nairobi (#3215) and the Liverpool School of Tropical Medicine (#15–005). This study is reported as per the Strengthening the Reporting of Observational studies in Epidemiology (STROBE) guidelines (S2 Table).

## Results

**Fig 2:**
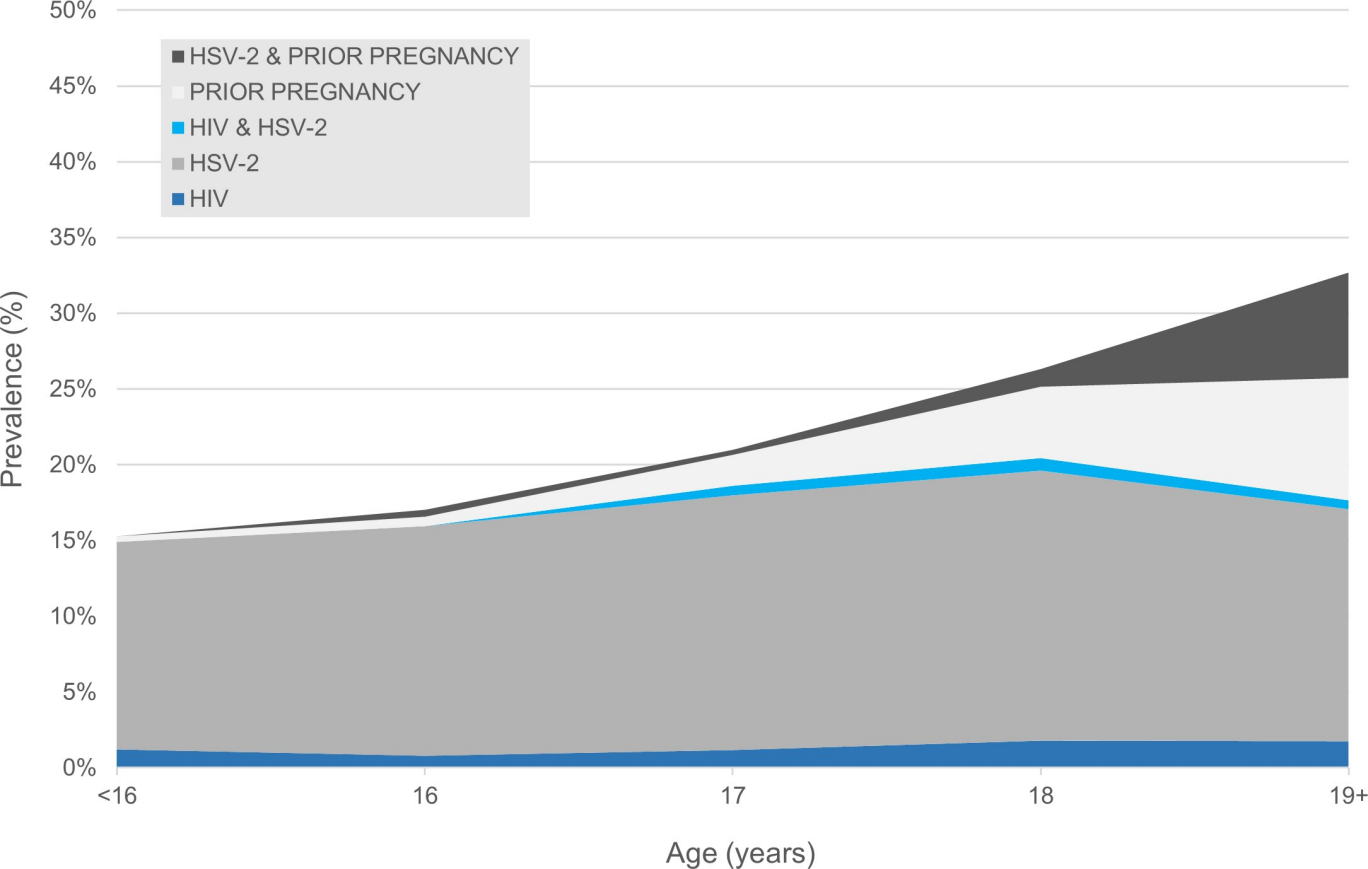
Combined baseline prevalence of HIV, HSV-2, and history of pregnancy among CCG girls (*n =* 3,998). CCG, Cups or Cash for Girls Trial; HIV, human immunodeficiency virus; HSV-2, herpes simplex virus type 2.

**Fig 3:**
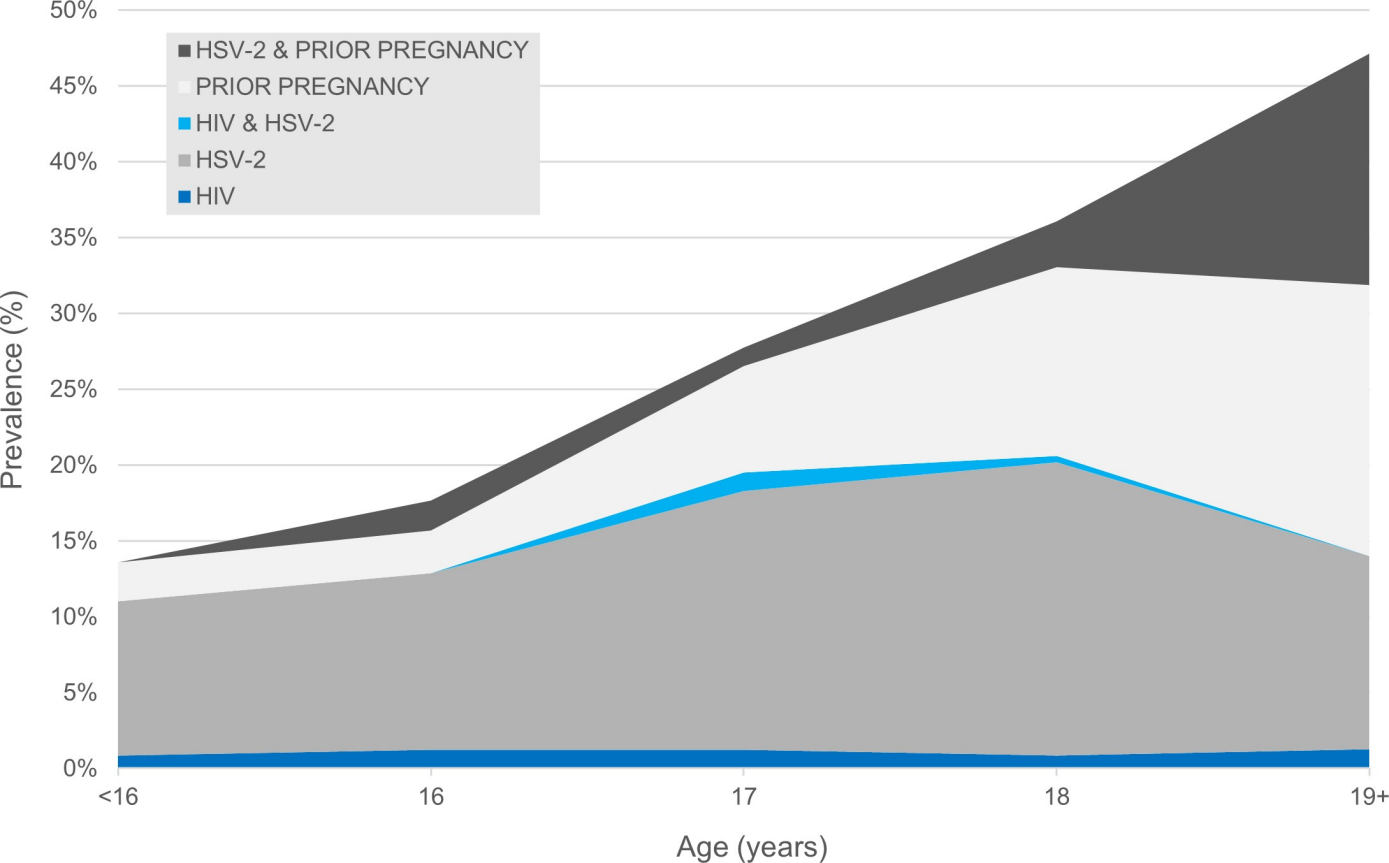
Combined baseline prevalence of HIV, HSV-2, and history of pregnancy among sexually active CCG girls (*n* = 1,090). CCG, Cups or Cash for Girls Trial; HIV, human immunodeficiency virus; HSV-2, herpes simplex virus type 2.

A total of 4,138 female students in secondary school class years Form 2 and 3 were enrolled between January 2017 and July 2018 across the 96 study schools; complete data and biomarkers were obtained for 3,998 girls. At enrollment, the median participant age was 17.1 years (interquartile range [IQR] 16.3 to 18.0 years; Table 1). The baseline prevalence of HIV was 1.7% (*n =* 66), 17.2% were HSV-2 seropositive (*n* = 686), 3.3% of girls reported having previously been pregnant (*n* = 133), and 27.3% of girls reported having previously had sex. Among sexually active girls, the prevalence of HIV was 1.9%, 19.2% were HSV-2 seropositive, and 12.2% had previously been pregnant; 31.8% of HIV positive girls and 30.5% of girls with HSV-2 reported sexual activity (Table 2). The combined prevalence of prior pregnancy, HIV, and HSV-2 rose from 15.3% in the youngest girls to 32.9% for those age 19 and older among all girls (Fig 2) and from 13.6% to 47.8% among the sexually active (Fig 3).

**Table 1:** Individual and household characteristics by SRH status among 3,998 adolescent girls attending secondary school, Siaya County, western Kenya, 2017–2018.

Individual and Household Characteristics	Total (*n =* 3,998)	Sexually active (*n =* 1,090)	History of pregnancy (*n* = 133)	HIV seropositive (*n* = 66)	HSV-2 seropositive (*n* = 686)
	N (%) or median (IQR)	N (%) or median (IQR)	N (%) or median (IQR)	N (%) or median (IQR)	N (%) or median (IQR)
Age (year)	17.1 (16.3–18.0)	17.5 (16.7–18.4)	18.6 (17.7–19.9)	17.7 (17.0–18.6)	17.3 (16.5–18.1)
Age categorical (year)*					
<16	759 (19.0)	118 (10.8)	3 (2.3)	9 (13.6)	104 (15.2)
16	1,134 (28.4)	249 (22.8)	12 (9.0)	9 (13.6)	177 (25.8)
17	1,124 (28.1)	328 (30.1)	28 (21.1)	21 (31.8)	200 (29.2)
18	612 (15.3)	233 (21.4)	36 (27.1)	16 (24.2)	121 (17.6)
19+	346 (8.7)	157 (14.4)	53 (39.8)	9 (13.6)	80 (11.7)
Age at menarche (year)	15.0 (14.0–15.0)	15.1 (14.0–15.0)	15.0 (14.0–15.0)	15.0 (14.0–15.5)	15.0 (14.0–15.0)
Early menarche (<13 years)	229 (5.7)	82 (7.5)	16 (12.0)	5 (7.6)	32 (4.7)
BMI					
Underweight (BMI <18.2)	212 (5.3)	40 (3.7)	5 (3.8)	7 (10.6)	29 (4.2)
Normal (BMI 18.2–25)	3,126 (78.2)	835 (76.6)	100 (75.2)	53 (80.3)	521 (75.9)
Overweight (BMI >25)	660 (16.5)	215 (19.7)	28 (21.1)	6 (9.1)	136 (19.8)
Marital status (MCW/SO)	262 (6.6)	88 (8.1)	9 (6.8)	7 (10.6)	52 (7.6)
Baby at home to care for	167 (4.2)	129 (11.8)	105 (78.9)	5 (7.6)	55 (8.0)
Orphan (no living parent)	135 (3.4)	39 (3.6)	6 (4.5)	8 (12.1)	25 (3.6)
Drinking (self-report)	15 (0.4)	9 (0.8)	0 (0.0)	1 (1.5)	1 (0.1)
Smoking (self-report)	8 (0.2)	2 (0.2)	0 (0.0)	0 (0.0)	0 (0.0)
SES categorical by quintile					
Quintile 1	801 (20.0)	246 (22.6)	40 (30.1)	14 (21.2)	144 (21.0)
Quintile 2	906 (22.7)	256 (23.5)	34 (25.6)	18 (27.3)	153 (22.3)
Quintile 3	695 (17.4)	184 (16.9)	21 (15.8)	9 (13.6)	115 (16.8)
Quintile 4	820 (20.5)	209 (19.2)	21 (15.8)	9 (13.6)	141 (20.6)
Quintile 5	776 (19.4)	195 (17.9)	17 (12.8)	16 (24.2)	133 (19.4)
SES (poorest vs less poor)	1,707 (42.7)	502 (46.1)	74 (55.6)	32 (48.5)	297 (43.3)
Work (last 7 days)	732 (18.3)	255 (23.4)	35 (26.3)	10 (15.2)	138 (20.1)
School absence during prior month due to work^±^	122 (3.1)	56 (5.1)	8 (6.0)	0 (0.0)	18 (2.6)
Have money saved	541 (13.5)	129 (11.8)	15 (11.3)	6 (9.1)	99 (14.4)
Received money from parents^†^	3,667 (91.7)	943 (86.5)	105 (78.9)	60 (90.9)	621 (90.5)
Received money from boyfriend/partner^†^	202 (5.1)	164 (15.0)	21 (15.8)	6 (9.1)	52 (7.6)
Received money from working^†^	560 (14.0)	228 (20.9)	37 (27.8)	8 (12.1)	100 (14.6)
Harassment for sex at school	473 (11.8)	245 (22.5)	14 (10.5)	7 (10.6)	70 (10.2)
Harassment for sex out of school	1,647 (41.2)	686 (62.9)	61 (45.9)	21 (31.8)	271 (39.5)
Happy at home (no)	283 (7.1)	123 (11.3)	28 (21.1)	6 (9.1)	48 (7.0)
Happy at school (no)	114 (2.9)	44 (4.0)	4 (3.0)	1 (1.5)	21 (3.1)
Overall well-being^0^	77.0 (65.2–78.3)	72.6 (60.9–73.9)	73.5 (60.7–73.9)	78.3 (65.2–77.7)	76.4 (65.2–78.3)
Low well-being	12 (0.30)	6 (0.6)	0 (0.0)	0 (0.0)	4 (0.6)
Moderately low well-being	255 (6.4)	110 (10.1)	18 (13.5)	3 (4.5)	49 (7.1)
Moderate well-being	1,504 (37.6)	490 (45.0)	53 (39.8)	27 (40.9)	252 (36.7)
High well-being	2,227 (55.7)	484 (44.4)	62 (46.6)	36 (54.5)	381 (55.5)
Used sanitary pads to manage entire period	2,417 (60.5)	617 (56.6)	70 (52.6)	44 (66.7)	401 (58.5)
Period stopped activities	1,096 (27.4)	408 (37.4)	45 (33.8)	10 (15.2)	192 (28.0)
Period severity					
Heavy	872 (21.8)	299 (27.4)	40 (30.1)	12 (18.2)	166 (24.2)
Normal	2,816 (70.4)	719 (66.0)	83 (62.4)	42 (63.6)	463 (67.5)
Light	310 (7.8)	72 (6.6)	10 (7.5)	12 (18.2)	57 (8.3)
Period duration					
<3 days	133 (3.3)	34 (3.1)	6 (4.5)	6 (9.1)	23 (3.4)
3–5 days	3,119 (78.0)	823 (75.5)	94 (70.7)	52 (78.8)	522 (76.1)
>5 days	666 (16.7)	209 (19.2)	32 (24.1)	8 (12.1)	129 (18.8)
Do not know	80 (2.0)	24 (2.2)	1 (0.8)	0 (0.00)	12 (1.7)
Missed school during period (all reasons)	604 (15.1)	247 (22.7)	29 (21.8)	6 (9.1)	97 (14.1)
Missed school due to period–most recent period (all reasons)	490 (12.3)	210 (19.3)	21 (15.8)	5 (7.6)	83 (12.1)
Subcounty					
Gem	801 (20.0)	246 (22.6)	40 (30.1)	14 (21.2)	144 (21.0)
Rarieda	906 (22.7)	256 (23.5)	34 (25.6)	18 (27.3)	153 (22.3)
Siaya	695 (17.4)	184 (16.9)	21 (15.8)	9 (13.6)	115 (16.8)
Ugenya	820 (20.5)	209 (19.2)	21 (15.8)	9 (13.6)	141 (20.6)
Ugunja	776 (19.4)	195 (17.9)	17 (12.8)	16 (24.2)	133 (19.4)

BMI, body mass index; IQR, interquartile range; MCW, married, cohabitating, widowed; PedsQL, Pediatric Quality of Life Inventory; SES, socioeconomic status; SO, single, other; SRH, sexual and reproductive health.

*23 girls were missing information on their age.

^±^Only girls reporting working responded on absence due to work.

^†^Girls could list more than one source of money.

^0^Overall well-being was measured through the 23-item PedsQL.

**Table 2:** Sexual risk and partner covariates by SRH status among 1,090 sexually active girls attending secondary school, Siaya County, western Kenya, 2017–2018.

Sexual and partner risk characteristics	Sexually active (*n =* 1,090)	History of pregnancy (*n =* 133)	HIV seropositive (*n* = 66)	HSV-2 seropositive (*n* = 686)
	N (%) or median (IQR)	N (%) or median (IQR)	N (%) or median (IQR)	N (%) or median (IQR)
Self-reported sex	1,090 (100.0)	133 (100.0)	21 (31.8)	209 (30.5)
Age at sexual debut (year)	16 (15.0–17.0)	16 (15.0–17.0)	15.5 (15.0–16.0)	16 (15.0–17.0)
Early sexual debut (<15)	147 (13.5)	12 (9.0)	3 (4.5)	20 (2.9)
First sex—force/rape	594 (54.5)	44 (33.1)	7 (10.6)	103 (15.0)
First sex—undesired	897 (82.3)	108 (81.2)	18 (27.3)	160 (23.3)
Touched indecently	419 (38.4)	47 (35.3)	14 (21.2)	110 (16.0)
Self-reported history of pregnancy	133 (12.2)	**-**	3 (4.5)	42 (6.1)
HIV seropositive	21 (1.9)	3 (2.3)	**-**	16 (2.3)
HSV-2 seropositive	209 (19.2)	42 (31.6)	16 (24.2)	**-**
Number of partners (6 months)^†^				
None	223 (20.5)	24 (18.0)	2 (9.5)	42 (20.1)
One	512 (47.0)	60 (45.1)	12 (57.1)	109 (52.2)
Two or more	175 (16.1)	15 (11.3)	6 (28.6)	30 (14.4)
Do not know	180 (16.5)	34 (25.6)	1 (4.8)	28 (13.4)
Number of lifetime partners^†^				
One	663 (60.8)	81 (60.9)	12 (57.1)	126 (60.3)
Two or more	281 (25.8)	42 (31.6)	6 (28.6)	57 (27.3)
Do not know	146 (13.4)	10 (7.5)	3 (14.3)	26 (12.4)
Age discordancy of partner^†^				
Younger than you	11 (1.0)	1 (0.8)	0 (0.0)	2 (1.0)
About the same age	447 (41.0)	50 (37.6)	11 (52.4)	86 (41.1)
Older than you by <5	174 (16.0)	33 (24.8)	5 (23.8)	38 (18.2)
Older than you by 5–9	61 (5.6)	12 (9.0)	1 (4.8)	10 (4.8)
Older than you by 10+	66 (6.1)	7 (5.3)	1 (4.8)	11 (5.3)
Do not know	331 (30.4)	30 (22.6)	3 (14.3)	62 (29.7)
Age discordancy (older)^†^	301 (27.6)	52 (39.1)	7 (33.3)	59 (28.2)
Was partner…^†^				
Someone you knew	854 (78.3)	119 (89.5)	19 (90.5)	170 (81.3)
Someone you did not know	236 (21.7)	14 (10.5)	2 (9.5)	39 (18.7)
If someone you knew…*				
Partner	642 (75.2)	98 (82.4)	15 (78.9)	143 (84.1)
Relative	58 (6.8)	5 (4.2)	1 (5.3)	8 (4.7)
Other	154 (18.0)	16 (13.4)	3 (15.8)	19 (11.2)
Partner circumcised^†^				
Yes	507 (46.5)	73 (54.9)	10 (47.6)	111 (53.1)
No	63 (5.8)	14 (10.5)	2 (9.5)	12 (5.7)
Do not know	520 (47.7)	46 (34.6)	9 (42.9)	86 (41.1)
Condom use (last 6 months)^†^	701 (64.3)	77 (57.9)	13 (61.9)	132 (63.2)
Family planning (any method)^†^	52 (4.8)	23 (17.3)	2 (9.5)	16 (7.7)
Engaged in sex for things or favours^0^	26 (9.3)	8 (19.1)	1 (9.1)	9 (8.0)
Money for sex with partner^‡^	67 (40.9)	9 (42.9)	2 (33.3)	17 (32.7)
Did something to get pads	255 (23.4)	41 (30.8)	5 (7.6)	103 (15.0)
Sex in exchange for pads	33 (3.0)	5 (3.8)	1 (1.5)	7 (1.0)

IQR, interquartile range; SRH, sexual and reproductive health.

^†^Partner characteristics were only collected for sexually active girls (*n* = 1,090; 133 pregnant girls; 21 HIV+ girls; and 209 HSV-2+ girls).

*Denominator is among girls whose first sex was with someone they knew.

^0^Only 684 girls responded to this question (281 sexually active girls, 42 girls with a history of pregnancy, 11 HIV+ girls, and 113 HSV-2+ girls).

^‡^Denominator is among girls responding “yes” to receiving money from partner (*n =* 202; 164 sexually active girls, 21 previously pregnant girls, 6 HIV+ girls, and 52 HSV-2+ girls).

### Sociodemographic and individual characteristics

Among the 3,998 participants, 6.6% reported being married or cohabiting with their partner, 4.2% reported taking care of a child at home, and 3.4% reported having no living parent (Table 1). Most girls’ BMI was normal (78.2%), with 5.3% classified as underweight and 16.5% as overweight. Menstrual characteristics reflected that 5.7% of girls started menstruating early, before age 13; 21.8% of girls experienced heavy bleeding during their period, with 16.7% bleeding for more than 5 days; and 27.4% reported being unable to participate in daily activities due to their period, including 12.3% who missed school during their most recent period. Low or moderately low well-being was reported by 6.7% of girls, 7.1% were not happy at home, and 2.9% were unhappy at school. Additionally, 41.2% of girls reported being harassed for sex outside of school, and 11.8% suffered the same at school; 14.6% of all girls reported having been touched indecently by a man or a boy (*n =* 582). Approximately 18.3% of girls reported performing nonschool-related work in the prior week, most commonly fetching water (90.8%) or working in the fields (68.4%); 30.5% of these girls reported missing school as a consequence.

As shown in Table 3, girls reporting sexual activity were older (aRR: 1.17 per additional year of age; CI 1.14 to 1.20, *p* < 0.001), had higher BMI, with 32.6% classified as overweight (aRR: 1.14; CI 1.02 to 1.28, *p* = 0.016 relative to girls with normal BMI), and were more likely to report their period stopped them from engaging in daily tasks (aRR: 1.14; CI 1.01 to 1.27, *p* = 0.032 post-adjustment for all 10 individual characteristics retained in the model). Harassment for sex both in school (aRR: 1.23; CI 1.09 to 1.38, *p* = 0.001) and out of school (aRR: 1.77; CI 1.57 to 2.00, *p* < 0.001) related to higher reported sexual activity. Sexual violence in the form of indecent touching yielded a 2.5-fold increase in sex (aRR: 2.52; CI: 2.26 to 2.81, *p* < 0.001). Girls receiving money from their boyfriend were 60% more likely to report sex than girls receiving money from other sources (aRR: 1.59; CI: 1.42 to 1.80, *p* < 0.001). Engaging in some nonschool-related work (aRR: 1.15; CI: 1.02 to 1.29, *p* = 0.020) and receiving money from working (aRR: 1.20; CI: 1.07 to 1.34, *p* = 0.002) were both associated with higher levels of reported sex among girls attending school, as was reporting having to “do things to acquire menstrual pads,” such as farm and housework, childcare, or engaging in sex for money, (aRR: 1.14; CI: 1.01 to 1.27, *p* = 0.032). In the univariate analysis (S3 and S4 Tables), being asked to give sexual favors, being married or cohabiting with a partner, and low household wealth were all shown to be associated with girls’ reported sex. While very few girls reported drinking alcohol (*n =* 15), it was significantly associated with higher rates of reported sex prior to adjustment. Similarly, menstrual related factors including using menstrual pads for their entire period, having heavier periods or periods of longer duration of bleeding, and missing school due to their periods were found to be related to reported sex prior to adjustment. Low well-being was associated with sexual activity (RR: 2.30; CI: 1.35 to 3.93, *p* = 0.002) but did not remain significant after adjustment.

**Table 3:** Relationship between individual risk factors and sexual activity among girls attending secondary school, Siaya County, western Kenya, 2017–2018.

*Among full cohort of 3*,*998 girls*	RR (95% CI)	*P* value	aRR* (95% CI)	*P* value
Age (year)	1.22 (1.19–1.26)	<0.001	1.17 (1.14–1.20)	<0.001
Early menarche (<13 years)	1.34 (1.12–1.61)	0.002	--	--
BMI				
Underweight (BMI <18.2)	0.71 (0.52–0.95)	0.022	0.93 (0.69–1.24)	0.600
Normal (BMI 18.2–25)	ref	ref	ref	ref
Overweight (BMI >25)	1.22 (1.10–1.35)	<0.001	1.14 (1.02–1.28)	0.016
HSV-2 seropositivity	1.15 (1.02–1.29)	0.023	--	--
Marital status (MCW/SO)	1.25 (1.03–1.52)	0.024	--	--
SES (poorest vs less poor)	1.16 (0.64–2.11)	0.6307	--	--
Drinking (self-report)	2.21 (1.54–3.18)	<0.001	--	--
Work (last 7 days)	1.36 (1.21–1.54)	<0.001	1.15 (1.02–1.29)	0.020
Received money from parents	0.58 (0.50–0.67)	<0.001	--	--
Received money from boyfriend/partner	3.33 (3.05–3.63)	<0.001	1.59 (1.42–1.80)	<0.001
Received money from working	1.62 (1.45–1.82)	<0.001	1.20 (1.07–1.34)	0.002
Touched indecently	3.67 (3.33–4.04)	<0.001	2.52 (2.26–2.81)	<0.001
Harassed for sex at school	2.16 (1.93–2.42)	<0.001	1.23 (1.09–1.38)	0.001
Harassed for sex out of school	2.42 (2.14–2.74)	<0.001	1.77 (1.57–2.00)	<0.001
Not happy at home	1.67 (1.47–1.90)	<0.001	--	--
Not happy at school	1.43 (1.16–1.77)	0.001	--	--
Overall wellbeing^0^			--	--
Low well-being	2.30 (1.35–3.93)	0.002		
Moderately–low well-being	1.98 (1.69–2.33)	<0.001		
Moderate well-being	1.50 (1.35–1.67)	<0.001		
High well-being	ref	ref		
Engaged in sex for things or favors	1.89 (1.53–2.33)	<0.001	--	--
Used sanitary pads to manage entire period	0.85 (0.77–0.94)	0.001	--	--
Did something to get pads	1.85 (1.64–2.09)	<0.001	1.14 (1.01–1.27)	0.032
Period stopped activities	1.58 (1.43–1.74)	<0.001	1.14 (1.04–1.25)	0.004
Period severity			--	--
Heavy	1.34 (1.19–1.51)	<0.001		
Normal	ref	ref		
Light	0.91 (0.73–1.14)	0.402		
Period duration			--	--
<3 days	0.97 (0.69–1.37)	0.857		
3–5 days	ref	ref		
>5 days	1.19 (1.07–1.33)	0.002		
Missed school due to period ever (all reasons)	1.65 (1.46–1.86)	<0.001	--	--
Missed school due to period–most recent period (all reasons)	1.32 (1.03–1.69)	0.026	--	--

aRR, adjusted risk ratio; BMI, body mass index; CI, confidence interval; HSV-2, herpes simplex virus type 2; MCW, married, cohabitating, widowed; PedsQL, Pediatric Quality of Life Inventory; RR, risk ratio; SES, socioeconomic status; SO, single, other.

Result considered statistically significant at *p* < 0.05.

*aRRs are adjusted for age, BMI, working in the last 7 days, receiving money from work, receiving money from a partner, being touched indecently, harassment for sex in school, harassment for sex out of school, reporting that they needed to do things to acquire pads, and reporting their menstrual period stopped them from completing daily tasks.

^0^Overall well-being was measured through the 23-item PedsQL.

Correlates related to history of pregnancy are presented in Table 4. After adjustment for the 8 risk factors retained in the model, girls who had been pregnant were significantly older (median 18.6 years; IQR 17.7 to 19.9 years) and age was strongly associated with having been pregnant prior to the study, reflecting a 56% increase in risk of adolescent pregnancy per year (aRR: 1.56; CI 1.44 to 1.69, *p* < 0.001). While not statistically significant after adjustment, low household wealth was associated with an increased risk of adolescent pregnancy: Girls in the lowest 2 quintiles had 38% increased risk of pregnancy (aRR: 1.38; CI 0.99 to 1.92, *p* = 0.055). Girls experiencing early menarche had twice the risk of having been pregnant (aRR: 2.05; CI 1.33 to 3.17, *p* = 0.001). Lower levels of harassment at school (aRR: 0.60; CI 0.36 to 0.98, *p* = 0.042) and happiness at home were detected among previously pregnant girls (aRR: 2.38; CI 1.51 to 3.74, *p* < 0.001); 81% of girls who had a live birth reported caring for their baby at home. Girls who reported doing things in exchange for menstrual pads such as farm and house work, childcare, or engaging in sex for money had 1.8 times elevated risk of having been pregnant (aRR: 1.81; CI 1.22 to 2.67, 0.003); and reporting being touched indecently was associated with 2.2 times the risk of having been pregnant (aRR: 2.29; CI: 1.62 to 3.24, *p* < 0.001). HSV-2–seropositive girls were 60% more likely to have been pregnant (aRR: 1.62; CI: 1.16 to 2.27, *p* = 0.005); HIV infection was not associated with pregnancy.

**Table 4:** Relationship between individual risk factors and adolescent pregnancy among girls attending secondary school, Siaya County, western Kenya, 2017–2018.

***Among full cohort of 3*,*998 girls***	**RR (95% CI)**	***P* value**	aRR* (95% CI)	***P* value**
Age (year)	1.54 (1.39–1.70)	<0.001	1.56 (1.44–1.69)	<0.001
Early menarche (<13 years)	2.25 (1.36–3.73)	0.002	2.05 (1.33–3.17)	0.001
SES (poorest vs less poor)	1.68 (1.19–2.38)	0.003	1.38 (0.99–1.92)	0.055
HSV-2 seropositivity	2.23 (1.59–3.11)	<0.001	1.62 (1.16–2.27)	0.005
Touched indecently	3.21 (2.19–4.69)	<0.001	2.29 (1.62–3.24)	<0.001
Harassed for sex at school	0.88 (0.51–1.51)	0.634	0.60 (0.36–0.98)	0.042
Not happy at home	3.50 (2.26–5.42)	<0.001	2.38 (1.51–3.74)	<0.001
Did something to get pads	2.70 (1.84–3.97)	<0.001	1.81 (1.22–2.67)	0.003
Engaged in sex for things or favors	4.36 (2.31–8.22)	<0.001	--	--
Received money from parents	0.34 (0.23–0.50)	<0.001	--	--
Received money from boyfriend/partner	3.52 (2.20–5.65)	<0.001	--	--
Received money from working	2.37 (1.63–3.44)	<0.001	--	--
***Among 1*,*090 sexually active girls***	**RR (95% CI)**	***P* value**	**aRR** ^‡^ **(95% CI)**	***P* value**
Age (year)	1.35 (1.24–1.47)	<0.001	1.33 (1.23–1.43)	<0.001
Early menarche (<13 years)	1.69 (1.07–2.69)	0.025	1.67 (1.20–2.34)	0.003
Household SES (poorest vs less poor)	1.47 (1.06–2.05)	0.020	1.36 (1.02–1.80)	0.034
Age at sexual debut (year)	1.18 (1.09–1.28)	<0.001	--	--
Early sexual debut (< 15 years)	0.47 (0.26–0.83)	0.009	--	--
First sex—not forced	2.43 (1.70–3.48)	<0.001	1.58 (1.11–2.24)	0.011
HSV-2 seropositive	1.95 (1.41–2.69)	<0.001	--	--
Harassed for sex at school	0.40 (0.23–0.69)	0.001	--	--
Harassed for sex out of school	0.49 (0.36–0.68)	<0.001	0.59 (0.44–0.79)	<0.001
Not happy at home	3.50 (2.26–5.42)	<0.001	1.71 (1.14–2.57)	0.009
Age discordancy of partner				
Younger than you	0.81 (0.12–5.45)	0.829	0.91 (0.12–6.66)	0.925
About the same age	ref	ref	ref	ref
Older than you by <5 years	1.71 (1.15–2.56)	0.009	1.84 (1.31–2.58)	<0.001
Older than you by 5–9 years	1.75 (1.12–2.75)	0.04	1.85 (1.15–2.99)	0.012
Older than you by 10+ years	0.95 (0.47–1.91)	0.877	1.27 (0.70–1.80)	0.633
Was partner…				
Someone you knew	2.36 (1.39–4.03)	0.002	1.74 (1.04–2.92)	0.035
Someone you did not know	ref	ref	ref	ref
Family planning (hormonal method)	4.15 (2.96–5.83)	<0.001	3.59 (2.70–4.77)	<0.001
Engaged in sex for things or favors	2.28 (1.17–4.43)	0.015	--	--
Did something to get pads	1.45 (0.98–2.14)	0.060	1.41 (1.00–1.97)	0.047

aRR, adjusted risk ratio; CI, confidence interval; HSV-2, herpes simplex virus type 2; RR, risk ratio; SES, socioeconomic status.

Result considered statistically significant at *p* < 0.05.

*aRRs are adjusted for age, early menarche, household SES, HSV-2 seropositivity, being touched indecently, being unhappy at home, and performing activities to acquire pads.

^‡^*For sexually active girls, aRRs are adjusted for age, early menarche, household SES, self-reporting that first sex was not forced, harassment for sex out of school, being unhappy at home, age discordancy of partner, whether their partner was someone they knew, using hormonal contraceptives, and performing activities to acquire pads.

When assessing predictors of HSV-2, girls’ age yielded a 7% annual increased risk in contracting HSV-2 in the multivariable analysis (aRR: 1.07; CI: 1.02 to 1.12, *p* = 0.003; Table 5), after adjusting for the 5 individual covariates retained in the model. Other factors related to HSV-2 in the multivariable analysis included age at first menstrual period—girls reaching menarche later had higher rates of HSV-2 (aRR: 1.08; CI: 1.02 to 1.15, 0.011); BMI—girls classified as overweight had higher risk of contracting HSV-2 compared to girls with normal body weight (aRR 1.24; 1.05 to 1.46, *p* = 0.010); history of pregnancy (aRR: 1.58; CI: 1.20 to 2.07, *p* = 0.001); and receiving money from a boyfriend (aRR 1.43; 1.13 to 1.80, *p* = 0.003). Girls taking care of a child at home had twice the prevalence of HSV-2 (RR: 2.00; CI 1.60 to 2.50, *p* < 0.001), although this variable was not included in the adjusted models due to collinearity with prior pregnancy experience.

**Table 5:** Relationship between individual and partner risk factors and HSV-2 seropositivity among adolescent girls attending secondary school, Siaya County, western Kenya, 2017–2018.

***Among full cohort of 3*,*998 girls***	**RR (95% CI)**	***P* value**	**aRR** * **(95% CI)**	***P* value**
Age (year)	1.13 (1.08–1.18)	<0.001	1.07 (1.02–1.12)	0.003
Age at menarche (year)	1.11 (1.03–1.18)	0.003	1.08 (1.02–1.15)	0.011
BMI				
Underweight (BMI <18.2)	0.82 (0.59–1.15)	0.251	0.89 (0.63–1.26)	0.524
Normal (BMI 18.2–25)	ref	ref	ref	ref
Overweight (BMI >25)	1.24 (1.04–1.46)	0.014	1.24 (1.05–1.46)	0.010
Received money from parents	0.86 (0.68–1.10)	0.229	--	--
Received money from boyfriend/partner	1.54 (1.22–1.95)	<0.001	1.43 (1.13–1.80)	0.003
Received money from working	1.05 (0.85–1.29)	0.661	--	--
Previous pregnancy	1.90 (1.48–2.42)	<0.001	1.58 (1.20–2.07)	0.001
Engaged in sex for things or favors	1.60 (0.99–2.60)	0.054	--	--
***Among 1*,*090 sexually active girls***	**RR (95% CI)**	***P* value**	**aRR** ^‡^ **(95% CI)**	***P* value**
Age (year)	1.16 (1.10–1.23)	<0.001	1.11 (1.04–1.20)	0.003
BMI				
Underweight (BMI <18.2)	0.42 (0.14–1.24)	0.116	0.41 (0.10–1.64)	0.207
Normal (BMI 18.2–25)	ref	ref	ref	ref
Overweight (BMI >25)	1.49 (1.15–1.92)	0.003	1.43 (1.09–1.88)	0.011
Early sexual debut (<15 years)	0.62 (0.40–0.96)	0.031	--	--
First sex—desired	1.42 (1.13–1.79)	0.003	1.27 (1.01–1.60)	0.043
First sex with someone known:				
Partner	1.75 (1.17–2.62)	0.007	1.64 (1.09–2.47)	0.019
Relative/other	ref	ref	ref	ref
Family planning (hormonal method)	1.81 (1.23–2.67)	0.003	1.72 (1.16–2.55)	0.007
Engaged in sex for things or favors	1.82 (1.01–3.29)	0.046	--	--

aRR, adjusted risk ratio; BMI, body mass index; CI, confidence interval; HSV-2, herpes simplex virus type 2; RR, risk ratio.

Result considered statistically significant at *p* < 0.05.

*aRRs are adjusted for age, age at menarche, BMI, receiving money from a partner, engaging in sex for things or favors, and reporting a prior pregnancy.

^‡^*For sexually active girls, aRRs are adjusted for age, BMI, self-reporting that they did not want to have sex at sexual debut, first sex being with someone they knew, using hormonal contraceptives, and engaging in sex for things or favors.

Age, BMI, orphanhood, and period-related covariates were associated with HIV in the multivariable analysis (Table 6), after adjusting for these same risk factors. Older girls were at heightened risk of HIV (aRR: 1.34; CI: 1.18 to 1.53, *p* < 0.001); and orphaned girls were nearly 3 times as likely to be HIV positive (aRR: 2.81; CI: 1.18 to 6.71, p = 0.020). Twenty HIV positive girls (30.3%) reported contracting the disease from their mothers at birth; data on source of HIV infection was only reported by 30 girls. Girls’ weight was negatively associated to HIV: underweight BMI reflected twice the risk of being HIV positive relative to normal weight girls (aRR: 2.07; CI: 1.00 to 4.30, *p* = 0.051) and 4.2 times relative to overweight girls (aRR 4.21; CI: 1.36 to 13.1, *p* = 0.013). Relating to menstruation, HIV positive girls had significantly lighter periods (aRR: 2.42; CI: 1.22 to 4.79, *p* = 0.012) and bled for fewer days (aRR: 2.81; CI: 1.16 to 6.82, *p* = 0.023) compared to girls with normal periods. These girls also reported less frequently that their period stopped them from engaging in daily tasks (RR: 0.43; CI 0.21 to 0.85, *p* = 0.016), possibly due to their less burdensome periods.

**Table 6:** Relationship between individual and partner risk factors and HIV seropositivity among adolescent girls attending secondary school, Siaya County, western Kenya, 2017–2018.

*Among full cohort of 3*,*998 girls*	RR (95% CI)	*P* value	aRR* (95% CI)	*P* value
Age (year)	1.28 (1.13–1.45)	<0.001	1.34 (1.18–1.53)	<0.001
BMI				
Underweight (BMI <18.2)	3.63 (1.20–10.99)	0.022	4.21 (1.36–13.1)	0.013
Normal (BMI 18.2–25)	1.87 (0.85–4.10)	0.120	2.03 (0.90–4.59)	0.087
Overweight (BMI >25)	ref	ref	ref	ref
Orphan (no living parent)	3.95 (1.74–8.95)	<0.001	2.81 (1.18–6.71)	0.020
Period stopped activities	0.47 (0.24–0.91)	0.026	0.43 (0.21–0.85)	0.016
Period severity				
Heavy	0.92 (0.51–1.68)	0.793	1.07 (0.56–2.04)	0.837
Normal	ref	ref	ref	ref
Light	2.60 (1.37–4.90)	0.003	2.42 (1.22–4.79)	0.012
Period duration				
<3 days	2.71 (1.18–6.23)	0.019	2.81 (1.16–6.82)	0.023
3–5 days	ref	ref	ref	ref
>5 days	0.72 (0.32–1.61)	0.424	0.68 (0.28–1.65)	0.396

aRR, adjusted risk ratio; BMI, body mass index; CI, confidence interval; RR: Risk ratio.

Result considered statistically significant at *p* < 0.05.

*aRRs are adjusted for age, BMI, orphanhood, reporting that their menstrual period stopped them from completing daily tasks, and the severity and duration of menstrual period.

### Participant and partner characteristics among girls reporting sexual activity

Among the 1,090 girls who reported having reached sexual debut at enrollment, the median age of first sex was 16 (IQR: 15 to 17), with 13.5% reporting initiating sex early, before age 15 (Table 2). Approximately 82.3% of sexually active girls declared they had not wanted to have sex (*n* = 897), and 54.4% declared their first sex was forced (*n* = 594). Girls mostly reported having a single sexual partner in the last 6 months (47.0%, *n* = 512), with 16.1% reporting 2 or more partners (*n* = 175); 25.8% of girls reported 2 or more lifetime sexual partners. Approximately 27.6% of girls reported that their sexual partner was older than them (*n =* 301). Approximately 21.7% of girls reported their first sexual partner was someone they did not know (*n =* 236), with this occurring significantly more often among girls who reported their first sex was forced (32.2% versus 9.1%; RR: 1.72, 1.56 to 1.90, *p* < 0.001). Among girls who knew their partner, nearly 1 in 4 girls reported that the individual was not their romantic partner, either a relative (6.8%) or other acquaintance (18%). Among 164 girls who received money from their boyfriend, 37.3% reported being asked for sex in return. Condom use in the past 6 months was reported by 73.2% of sexually active girls. A total of 52 girls reported using hormonal contraception, with the implant cited as the most common method (50.0%).

After adjusting for 10 associated sexual risk factors, in the multivariable analysis, heightened risk of pregnancy was found in sexually active girls who were older (aRR: 1.33; CI: 1.23 to 1.43, *p* < 0.001; Table 3), belonged to the poorest 2 wealth quintiles (aRR: 1.36; CI: 1.02 to 1.80, *p* = 0.034), and had experienced early menarche (aRR: 1.67; CI: 1.20 to 2.34, *p* = 0.003). Those who had experienced a prior pregnancy reported lower rates of harassment for sex outside of school (aRR: 0.59; CI: 0.44 to 0.81, *p* = 0.001) and were less happy at home (aRR: 1.71; CI: 1.14 to 2.57, *p* = 0.009). Girls reporting their first sex was not forced were more likely to report prior pregnancy (1.58; CI: 1.11 to 2.24, *p* = 0.011). Girls on hormonal contraceptives were 3.5 times as likely to have been previously pregnant (aRR: 3.59; CI: 2.70 to 4.77, *p* < 0.001). Partner characteristics associated with adolescent pregnancy after adjustment against all other covariates retained in the model included the girl knowing her partner at first sex (aRR: 1.74; CI: 1.04 to 2.92, *p* = 0.035; Table 3) and the partner being older than her (aRR: 1.84; CI: 1.31 to 2.58, *p* < 0.001 for 0 to 5 years older and aRR: 1.85; CI: 1.15 to 2.99, *p* = 0.012 for 5 to 10 years relative to same-age partners). Girls’ reports of having to do things in exchange for pads were also related to higher pregnancy risk (aRR: 1.41; CI: 1.00 to 1.97), as was girls reporting engaging in transactional sex for goods or favors; however, the latter was not significant after adjustment.

Sexually active girls had higher rates of HSV-2 at 19.2% (RR: 1.15; CI: 1.02 to 1.29, *p* = 0.023; Table 5). In the models adjusted for all 5 associated covariates, reporting that sex was not forced at sexual debut was associated with HSV-2 acquisition (aRR: 1.27; CI: 1.01 to 1.60, *p* = 0.043). Girls reporting their first sex to be with someone they considered a romantic partner were more likely to have contracted HSV-2 (aRR: 1.64; CI: 1.09 to 2.47, *p* = 0.019), and those reporting using hormonal contraceptives were more likely to be HSV-2 seropositive (aRR: 1.72; CI: 1.16 to 2.55, *p* = 0.007). HIV was not found to be associated with any individual or partner risk behaviors among sexually active girls.

## Discussion

This study presents biological and survey data collected at baseline among girls attending secondary school who were enrolled in a large cluster randomized controlled trial in rural western Kenya and identifies the socioeconomic and behavioral correlates related to girls’ sexual exposure, risk of early pregnancy, and acquisition of HIV or HSV-2. Our study underscores girls’ vulnerability to sexual harassment and coercion in our study area. At trial enrollment, over 1 in 4 girls reported sexual debut, 1 in 6 had already contracted HSV-2, and, among sexually active girls, 1 in 8 was already a mother. Baseline prevalence of HIV was low (1.7%) and in line with declining estimates previously reported in the area [51]. Orphaned girls were nearly 3 times as likely to be HIV positive. Girls who were underweight had a 2-fold higher risk of being HIV positive relative to normal weight girls and a 4.5-fold higher risk relative to overweight girls. Girls with abnormal periods were also more likely to be HIV positive, with 2.4 times the risk of HIV among girls reporting light bleeding and a 2.8-fold risk among those reporting bleeding for fewer than 3 days. Sexual activity was elevated in girls who were older, overweight, experiencing harassment for sex in school and out of school, and those reporting working for pay; it was 2.5-fold higher in girls reporting being touched indecently. Pregnancy risk was most elevated in older girls, those from lower-income households, and those who were HSV-2 seropositive. Early menarche was associated with a 2-fold increase in adolescent pregnancy, and these girls were 2.5 times as likely to report being unhappy at home. HSV-2 seropositivity was highest among older girls, those who reached menarche later, were overweight, or received money from boyfriends. Girls who were previously pregnant were 1.6 times more likely to be HSV-2 seropositive. Among sexually active girls, pregnancy and HSV-2 seropositivity were also more commonly seen in girls whose first sex was not forced and was desired and among those who knew their partner at first sex. Girls who were on hormonal contraceptives were 3.5 times as likely to have previously been pregnant and 1.7 times as likely to have HSV-2. Girls with older partners were nearly twice as likely to have been pregnant.

In our study, early age at menarche and higher body mass were closely related to girls’ SRH outcomes. Girls experiencing menarche prior to age 13 were more likely to have been previously pregnant, while those with higher-than-normal BMIs had higher levels of sex and HSV-2 seropositivity. Early age at menarche has come into focus as an important pubertal factor affecting girls’ vulnerability to SRH harms [52]. Our findings are aligned with studies in LMICs showing early menarche to be associated with earlier sexual initiation, early pregnancy, and certain STIs including HSV-2 [52]. While evidence from high-income countries has indicated decreasing adolescent sexual activity for incremental increases in BMI [53] (assumedly due to the importance of slimness for romantic success), our study indicates that this association does not hold in settings like rural western Kenya. While limited data on adolescent BMI exist in these contexts, it has been suggested that being underweight in these areas is perceived as not having good health (particularly in HIV endemic regions), or being physically immature [54]. While the interrelationship between both pubertal markers (early menarche and BMI) is still being established, evidence to date suggests that elevated BMI at young ages leads to faster growth velocity and earlier menarche among adolescents [47,55,56], possibly highlighting the crucial role proper nutrition plays in girls’ long-term SRH. Our study adds to this evidence and supports interventions targeting girls who reach puberty early.

Our study found that adolescent girls experienced high levels of sexual pressure and coercion in these settings; over 1 in 7 girls reported being touched indecently by a man (14.6%), and over half of sexually active girls reported they had not engaged in sex by their own volition, reporting instead that they had been tricked or forced. A higher proportion still (82%) reported that, while they had agreed, they had not wanted to have sex at the time of first sex. Sexual harassment both inside and outside of school was common and significantly related to sexual debut among girls in school. Our study adds to the body of evidence confirming that harassment, trickery, and force are common [4,25,57]. Wider studies show girls facing sexual abuse during adolescence obtain less education and lower lifetime earnings and can face serious adverse health outcomes such as depression and severe anxiety, illicit drug use, and STIs [58,59]. In our study, poor mental well-being was also associated with elevated levels of sexual activity. Poorer well-being has also been reported among adolescents who feel unsafe at school or fear violence [60].

Lower levels of harassment were related to higher prevalence of prior pregnancy among adolescent girls attending secondary school. While the discourse around adolescent childbearing predominantly highlights the negative ramifications of early pregnancy (i.e., maternal health complications, school dropout, intergenerational transmission of poverty, and lower economic productivity), some literature from these contexts has indicated that motherhood makes some girls happy and allows them some security and stability as they navigate entering adulthood [61,62]. Given the cross-sectional nature of our study, the causal relationship of the association measured in our study could be that early pregnancy and childbearing lead to lower levels of harassment among girls in school. However, lower levels of happiness at home were also reported; thus, the social desirability of childbearing needs to be better understood to construct effective approaches to tackling adolescent pregnancy.

Our findings support previous studies that suggest that low SES is a constant predisposing factor for adolescent pregnancy globally as well as in East Africa [63,64]. Evidence out of western Kenya has shown that girls pursuing educational opportunities generally come from households of higher SES than those out of school and that retention in school protects against early pregnancy [6,65]. Our study supports this body of evidence by again implying that low SES may independently create conditions that continue to predispose girls to adolescent pregnancy and that added focus must be given to girls living in low SES households regardless of their schooling status. Adolescent pregnancy was also correlated with HSV-2 seropositivity. Some literature has suggested that, due to its high prevalence, cumulative probability, and strong positive predictive value, HSV-2 can be used as a proxy indicator for sexual activity [20,66,67]. We anticipated seeing high concordance between girls who were sexually active and those who were HSV-2 seropositive, but approximately 70% of HSV-2–seropositive girls reported never engaging in sex, possibly indicating higher than anticipated nonsexual transmission of HSV-2 [67]. Some studies have reported high levels of child autoinoculation and caregiver transmission occurring during activities such as bathing and toileting [67], suggesting that more study is warranted on the HSV-2 transmission potentials of nonpenetrative sex in these younger cohorts. If used as a marker to validate girls’ self-reported accounts of sexual debut, sexual activity in our cohort would increase, indicating that these are conservative estimates of the true prevalence of sexual activity among adolescent girls attending secondary school in western Kenya. Other studies in Kenya have also identified inconsistent sexual histories reported by adolescent girls [68], and high discordance between biomarkers and self-reports of having sex [22,69], recommending the use of STI biomarkers to improve the validity of SRH findings in trials targeting adolescents. Our study supports the need for collecting biological data for accurate measurement.

Contraceptive use was low and associated with higher rates of pregnancy and HSV-2, in line with other studies on adolescents in western Kenya reporting that use of family planning is uncommon [36,65]. In relation to pregnancy, it is possible that girls who had become pregnant accessed health services postpartum that provided contraceptives to reduce the chances of repeat pregnancy. As related to HSV-2, evidence is growing, which suggests that hormonal contraceptives may change the genital tract flora or vaginal epithelial structure and lead to heightened susceptibility to STIs [70]. While adequate family planning is critical for adolescents at risk of early pregnancy, STI protective measures like condom use might be best promoted in HIV and HSV-2 endemic areas where barrier methods are required to prevent the spread of disease. Studies have also noted a strong effect on HIV caused by HSV-2 seropositive status [71], highlighting that efforts to reduce HSV-2 transmission may have positive spillover effects for HIV.

Our study also highlighted that correlates of HIV acquisition appeared unrelated to the other SRH outcomes in this population. HIV seropositivity was significantly associated with orphanhood status (i.e., no living parent) and a considerable number of girls reported they had contracted the disease at birth. As previously reported in this study area [69], mother-to-child transmission of HIV continues to be prevalent [72], and our study corroborates the occurrence of perinatal infections in this HIV endemic region. While some evidence suggests that orphans are highly vulnerable to contracting HIV, the causal pathway is mediated by schooling (with higher school dropout among orphans placing them at heightened risk of HIV [73]), a reality not played out in our school-going population.

Girls’ nutritional status was highly associated with HIV seropositivity: Girls with lighter periods of shorter duration and underweight girls were more likely to be HIV positive. Although evidence on menstrual disturbances in HIV positive women is inconclusive, limited evidence has shown that HIV serostatus increased the odds of having a very short menstrual cycle [74], as well as conditions like amenorrhea, which is characterized by very light to nonexistent periods [75,76]. These associations are stronger among HIV-infected women with higher weight loss [75]. It has separately been reported that HIV-infected women have lower BMIs. Our study adds to this body of evidence, indicating that, in our adolescent population, HIV-infected girls may be experiencing early symptoms of anovulation and progressive nutritional deficits.

Limitations of the study are noted. Firstly, given the cross-sectional nature of the data, temporality could not be established on cause or effect of risk factors related to girls’ SRH outcomes, making it possible that the causal direction of some associations, such as pregnancy and happiness at home, work in reverse. Nonetheless, the associations presented here are primarily to understand the demographic profile of girls at heightened risk and generate hypotheses of causal pathways that will be evaluated throughout the course of the larger trial; the selection of covariates followed standard epidemiological practice as described in the methods, and all analyses presented here were planned prior to survey development. Secondly, all behavioral data were self-reported including measures related to partners. We note underreporting of stigmatized risk factors and outcomes; for example, 34.8% of lab-confirmed HIV positive girls self-reported being HIV negative in the survey, and the low self-reported sexual activity among HSV-2–seropositive girls suggests possible social desirability bias in the girls’ reported responses, which has also been noted in other studies among adolescent populations [25,68,70,77]. This phenomenon may also play a role in the low rates of sexual activity reported by married, cohabiting, and widowed girls; Cho and colleagues found that 65% of married adolescents reported no sexual activity, suggesting that girls may have misreported their marital status or, conversely, felt unwilling to disclose information about sexual activity during a school-based survey when abstinence is promoted through the curriculum [69]. We note also that pregnancy tests were not conducted to limit risk of school expulsion due to pregnancy disclosure, thus history of pregnancy may have also been underreported as a result. Thirdly, a small percentage of HSV-2 results (3.4%) yielded an indeterminate result; these were reclassified as negative, possibly underestimating the true HSV-2 positivity rate in the population. Fourthly, many studies have noted that girls attending secondary school are a select population of adolescents who are less vulnerable to SRH harms than girls who are out of school [6,8]. Additionally, our inclusion criteria and selection of schools further limited our population to menstruating public school day scholars. Thus, we note that our findings may not be generalizable to all adolescents in these settings. Lastly, as part of a larger trial with specific objectives, some of our variables are limited in their detail, restricting interpretation.

## Conclusions

Our findings indicate that girls’ individual and household level factors are significantly associated with their SRH during adolescence. Adolescence is a critical time in which girls face heightened sexual pressures that may lead to risky behaviors with lasting consequences. We find that young girls in Kenya face high risks of early pregnancy and HSV-2 acquisition. Girls’ low reported contraceptive use suggests that access to family planning prepartum and/or self-perceived risk of pregnancy is poor in these areas and that educational programs and adolescent-focused service provision are warranted. As age of menarche drops globally, girls’ vulnerability to risky sexual behaviors and adverse SRH outcomes may be increasing. Additionally, as under and overnutrition become increasingly coexistent in these settings, understanding adolescents’ nutritional status as they transition into adulthood is relevant to SRH programming. Appropriate educational programs and interventions tackling adolescent girls SRH are needed.

## Supporting information

S1 Survey Baseline study questions from the Cups or Cash for Girls Trial sociobehavioral questionnaire. (PDF)

S1 Fig Distribution of BMI among 3,998 adolescent girls attending secondary school, Siaya County, western Kenya, 2017–2018. BMI classifications were based on percentile ranks: “underweight” classified as BMI <18.2, “normal weight” as BMI 18.2–25, and “overweight” as BMI >25. BMI, body mass index.
(TIF)

S2 Fig Distribution of household wealth among 3,998 adolescent girls attending secondary school, Siaya County, western Kenya, 2017–2018. Absolute index based on girls reported household assets (Kabudula, 2017): Red reference line separates girls in households designated to the bottom 2 wealth quintiles from those living in households in the top 3 quintiles.
(TIF)

S1 Table Stepwise model variable selection check via “swboot” bootstrap replications. Bootstrapped replications using STATA swboot were performed to validate variable selection of stepwise models; bold indicates concordance with stepwise models, while (*) indicates a deviation from stepwise model. ^0^Overall well-being was measured through the 23-item Pediatric Quality of Life Inventory (PedsQL).
(DOCX)

S2 Table Strengthening the Reporting of Observational studies in Epidemiology (STROBE) Checklist. (PDF)

S3 Table Univariate associations between individual and partner risk factors and outcomes of interest among 3,998 adolescent girls in secondary school, Siaya County, western Kenya, 2017–2018. CI, confidence interval; MCW, married, cohabitating, widowed; RR, risk ratio; SO, single, other. †Girls could list more than one source of money. ^0^Overall well-being was measured through the 23-item Pediatric Quality of Life Inventory (PedsQL).
(DOCX)

S4 Table Univariate associations between sexual risk factors and outcomes of interest among 1,090 sexually active adolescent girls attending secondary school, Siaya County, western Kenya, 2017–2018. CI, confidence interval; RR, risk ratio.
(DOCX)

## Abbreviations

aRR: adjusted risk ratio
BMI: body mass index
CCG: Cups or Cash for Girls Trial
CI: confidence interval
GLM: generalized linear model
HSV-2: herpes simplex virus type 2
IQR: interquartile range
LMICs: low- and middle-income countries
RR: risk ratio
SES: socioeconomic status
SRH: sexual and reproductive health
STI: sexually transmitted infection
VIF: variance inflation factor
