# Supplementary material for: Factors associated with the prevalence of HIV, HSV-2, pregnancy, and reported sexual activity among adolescent girls in rural western Kenya: A cross-sectional analysis of baseline data in a cluster randomized controlled trial
Source: PLoS Med. 2021 Sep 28;18(9):e1003756. doi: 10.1371/journal.pmed.1003756 (PMC8478198; doi:10.1371/journal.pmed.1003756)
Supplement: S1 Survey — (PDF) [file pmed.1003756.s001.pdf]

Data Dictionary – Baseline Cups or Cash for Girls Trial sociodemographic and quality of life survey

Variable Name	Question Text	Saved Value
bqdate	Date of Interview	User selected date
bqa1	What is your main source of water at home†	1 Pond<br>2 Borehole<br>3 Lake<br>4 Pipe in house<br>5 Rainwater<br>6 River<br>7 Stream<br>8 Other
bqa1_oth	Specify:	User entered text
bqa2	What type of latrine do you use at home?	1 Flush toilet<br>2 Bush/field<br>3 Traditional pit<br>4 Ventilated improved pit (VIP) latrine<br>5 Other
bqa2_oth	Specify:	User entered text
bqa3	What is used for light in your house?	1 Electricity<br>2 Kerosene<br>3 lampTin lamp<br>4 Candles<br>5 Other
bqa3_oth	Specify:	User entered text
bqa4	What type of flooring material do you have at your house?	Earth/sand<br>2 Cement<br>3 Dung<br>4 Ceramic tiles<br>5 Other
bqa4_oth	Specify:	User entered text
bqa5	What type of roofing material do you have at your house†	1 Straw<br>2 Tin/Iron<br>3 SheetsTiles<br>4 Other
bqa5_oth	Specify:	User entered text
bqa6	Where does your family cook at home†	1 Inside the house<br>2 In a separate building<br>3 Outdoors<br>4 Other
bqa6_oth	Specify:	User entered text

Data Dictionary – Baseline Cups or Cash for Girls Trial sociodemographic and quality of life survey

Variable Name	Question Text	Saved Value	
bqa7	What type of cooking fuel does your family use†	1<br>2<br>3<br>4<br>5	Wood<br>Charco<br>al<br>Gas cylinders<br>Electricity<br>Other
bqa7_oth	Specify:	User entered text	
bqa8	Do you or a family member in your household have a mobile phone†	 1	No<br>Yes
bqa9	Is there a TV in your household?	0<br>1	No<br>Yes
bqa10	Is there a radio in your household?	0<br>1	No<br>Yes
bqa13	What is your father's occupation, that is what kind of work does he mainly do?	1<br>2<br>0<br>98<br>99	Farmer/Shamba<br>Other<br>Does not work<br>Father not alive<br>Don't know
bqa15	What is your mother's occupation, that is what kind of work does she mainly do?	1<br>2<br>0<br>98<br>99	Farmer/Shamba<br>Other<br>Does not work<br>Mother not alive<br>Don't know
bqa15_oth	Specify:	User entered text	
bqa16	Is your mother paid in cash or in kind for this work or i• she not paid at all	1<br>2<br>3<br>4<br>99	Cash<br>In kind<br>Cash and in kind<br>Not paid<br>Don't know
bq1	What is your marital status? Cohabiting means that you are living with your partner with no formal marriage certificate.	 2<br>3<br>4<br>5	Single<br>Married<br>Cohabiting<br>Widowed<br>Other

Data Dictionary – Baseline Cups or Cash for Girls Trial sociodemographic and quality of life survey

Variable Name	Question Text	Saved Value	
bq5	Do you have a baby or child you take care of?	0	No
		1	Yes
bq6	Who is the birth mother of this child†	1	Me
		2	My partner other wife
		3	My mother
		4	A relative in the household
		5	A relative not in the household
		6	Other
bq7	Are you normally happy at home?	0	Not happy
		1	Just OK
		2	Happy
bq8	Are you normally happy at school?	0	Not happy
		1	Just OK
		2	Happy
bq9	During the last month that your classmates were attending school, did you miss any time at school?	0	No
		1	Yes
bq10	If yes, how many days of school did you miss in the	User entered integer	
bq12	Do you have good friends at school?	0	No
		1	They are just ok
		2	Yes
bq13	How much do you like being at school'7	0	Do not like it
		1	It is just ok
		2	Like it
bq14	Do girls in your school drink alcohol?	0	None
		1	Some of them
		2	Many
		99	Don't know
bq15	Do girls in your school smoke cigarettes?	0	None
		1	Some of them
		2	Many
		99	Don't know
bq16	If there are any boys in your school, do they drink alcohol?	0	None
		1	Some of them
		2	Many
		98	No boys; girls school only
		99	Don't know

Data Dictionary – Baseline Cups or Cash for Girls Trial sociodemographic and quality of life survey

Variable Name	Question Text	Saved Value	
bq17	Do boys in your school smoke cigarettes†	0<br>1<br>2<br>98<br>99	None<br>Some of them<br>Many<br>No boys; girls school only<br>Don't know
bq18	Do you drink alcohol?	0<br>1<br>2	Never<br>Sometimes<br>Regularly
bq19	Do you smoke cigarettes?	0<br>1<br>2	Never<br>Sometimes<br>Regularly
bq23	In the past 6 months: Have you been touched indecently by a boy/man?	0<br>1<br>2<br>3	Never<br>Just once<br>A few times<br>Many times
bq32	When inside the school, do boys or men harass girls for sex?	 1<br>2	Never<br>A few times<br>Many times
bq34	When outside the school, do boys or men harass girls for sex?	o<br>1<br>2	Never<br>A few times<br>Many times
bq36	Have you ever had sex with a man or boy?	0<br>1<br>2<br>3	Never<br>Just once<br>A few times<br>Many times
bq37	Has a man or boy ever forced or threatened to make you have sex?	0<br>1<br>2<br>3	Never<br>Just once<br>A few times<br>Many times

Data Dictionary – Baseline Cups or Cash for Girls Trial sociodemographic and quality of life survey

Variable Name	Question Text	Saved Value	
bq39	Do you remember how old you were when you first had sex or were forced or threatened into having sex?	o	No
		1	Yes
bq40	What age were you when you first had sex or were forced or threatened into having sex?	User entered integer	
bq41	Do you remember in which year or month you first had sex or were forced or threatened into having sex?	o	No
		1	Yes
bq42_y	Enter 4 digit year	User entered integer	
bq42_m	Enter month number	User entered integer	
bq43	The first time you had sex (or were forced or threatened to make you have sex with) a boy or man, how old was the man or boy? Was he…	1	Younger than you
		2	About the same age
		3	Older than you by less than 5 years
		4	Older than you by 5 - 9 years
		5	Older than you by 10 years or more
		99	Don't know
bq44	The first time you had sex (or were forced or threatened to make you have sex with) a boy or man,who was the man or boy? Was he…	o	Someone you had never seen before
		1	Someone you knew
bq45	If you did know him, was he…		Boyfriend but not living with him
		2	Husband but not living with him
		3	Partner/husband after living together
		4	Relative
		5	Other person
bq46	The first time you had sex (or were forced or threatened to make you have sex with) a boy or man, did you want to have sex with this man or boy?	0	No
		1	Yes
bq47	The first time you had sex (or were forced or threatened to make you have sex with) a boy or man, was he circumcised or uncircumcised?	1	Circumcised
		2	Uncircumcised
		3	Don't know
bq48	How many boys or men have you had sex with (or been forced or threatened to make you have sex with)in your life? It's ok if you can't remember?	User entered integer	
bq50	Have you or your partner ever used condoms to delay or avoid a pregnancy or sexually transmitted infection?	o	No
		1	Yes

Data Dictionary – Baseline Cups or Cash for Girls Trial sociodemographic and quality of life survey

Variable Name	Question Text	Saved Value	
bq61	Does the boy or man give you something for having sex with him?	o<br>1	No<br>Yes
bq62_q	If yes, what?	For display purpose only	
bq62_1	Money	0<br>1	No<br>Yes
bq62_2	Food/drink	0<br>1	No<br>Yes
bq62_3	House items	0<br>1	No<br>Yes
bq62_4	School items	0<br>1	No<br>Yes
bq62_5	Help with exams	0<br>1	No<br>Yes
bq62_6	Pads for monthly period	0<br>1	No<br>Yes
bq62_7	Less beatings/bad things	0<br>1	No<br>Yes
bq62_8	Clothes/things	0<br>1	No<br>Yes
bq62_9	Other	0<br>1	No<br>Yes
bq63	Are you currently using any family planning methods?	1<br>0	Yes<br>No method

Data Dictionary – Baseline Cups or Cash for Girls Trial sociodemographic and quality of life survey

Variable Name	Question Text	Saved Value	
bq63_1_q	If yes, which family planning method are you using?	For display purpose only	
bq63_1_0	No method	0	No
		1	Yes
bq63_1_1	Birth control pills	0	No
		1	Yes
bq63_1_2	Injection	0	No
		1	Yes
bq63_1_3	Implant	0	No
		1	Yes
bq63_1_4	Abstinence	0	No
		1	Yes
bq63_1_5	Other	0	No
		1	Yes
bq63_1_oth	You chose other, please specify which type of family planning method you are using?	User entered text	
bq64	Are you currently pregnant?	0	No
		1	Yes
bq65	Are you currently trying to get pregnant?	0	No
		1	Yes
bq66	Have you ever been pregnant'7	0	No
		1	Yes
bq67	How many times have you been pregnant'7	User entered integer	
bq68	How many babies have you given birth to?	User entered integer	
bq69	What happened with the most recent pregnancy?	1	Had a miscarriage
		2	Aborted/terminated
		3	Baby born but died
		4	Baby born alive
		5	Currently still pregnant
		6	Other
bq70	If "baby born alive," who does this baby live with'7	1	Me
		2	Not me

Variable Name	Question Text	Saved Value	
bq71	If "not me" then who?	1	My parents
		2	My sister or brother
		3	Other family
		4	The child's father
		5	The father's family
		6	Other
bq73	In the past 6 weeks did you have a period†	0	No
		1	Yes
bq74	Have you ever used sanitary pads?	0	No
		1	Yes
your_recent_period	All the next questions are for your recent period in school term-time	For display purpose only	
bq77	Did you use any sanitary pads to help manage your period?		Yes; entire period
		2	Yes; part of period
		0	No
bq83	How many days did you bleed during your most recent period?	User entered integer	
bq84	Was it heavy, normal or light†	1	Heavy
		2	Normal
		3	Light
bq85	Did your period stop you from doing things?	0	No
		1	Yes
bq86_q	If yes, what did you stop doing?	For display purpose only	
bq86_1	Attending school	0	No
		1	Yes
bq86_2	Sports at school	0	No
		1	Yes
bq86_3	Work at school	0	No
		1	Yes
bq86_4	School homework assignments	0	No
		1	Yes
bq86_5	Other work outside of school	0	No
		1	Yes
bq86_6	Other	0	No
		1	Yes

Data Dictionary – Baseline Cups or Cash for Girls Trial sociodemographic and quality of life survey

Variable Name	Question Text	Saved Value	
bq92	Did you miss school (during recent period in term time)?	0<br>1	No<br>Yes
bq93	If yes, did you miss because of something to do with your period?	o<br>1	No<br>Yes
bq96	During recent period, did you have to do things to get pads or other menstrual items?	o<br>1	No<br>Yes
bq97_q	If yes, what?	For display purpose only	
bq97_1	Laundry	0<br>1	No<br>Yes
bq97_2	Childcare	0<br>1	No<br>Yes
bq97_3	Housework	0<br>1	No<br>Yes
bq97_4	Shamba	0<br>1	No<br>Yes
bq97_5	Bar work	0<br>1	No<br>Yes
bq97_6	Sex	0<br>1	No<br>Yes
bq97_7	Other	0<br>1	No<br>Yes
bq98	Apart from schoolwork, do you have other tasks or work you must do?	0<br>1	No<br>Yes
bq99_q	Tick any of the below that you did last month:	For display purpose only	
bq99_1	Household chores	0<br>1	No<br>Yes
bq99_2	Look after siblings	0<br>1	No<br>Yes
bq99_3	Look after other family	0<br>1	No<br>Yes
bq99_4	Laundry/mend clothes	0<br>1	No<br>Yes
bq99_5	Work in the shamba	0<br>1	No<br>Yes
bq99_6	Fetch water	0<br>1	No<br>Yes

Data Dictionary – Baseline Cups or Cash for Girls Trial sociodemographic and quality of life survey

Variable Name	Question Text	Saved Value	
bq99_7	Help in shop or business	0<br>1	No<br>Yes
bq99_8	Work in bar	0<br>1	No<br>Yes
bq99_9	Sex work	0<br>1	No<br>Yes
bq99_10	Other things	0<br>1	No<br>Yes
bq100	Yesterday, how many hours did you spend doing household chores, such as cooking, cleaning, laundry, collecting firewood, water?	User entered decimal	
bq101	Aside from schoolwork and housework, have you done any work in the last seven days?	0<br>1	No<br>Yes
bq102	If yes, what?	User entered text	
bq103	As you know, some girls take up jobs for which they are given pocket money or gifts. Others sell things, have a small business or work on the family farm or in the family business. Have you ever done any chores or activities for which you got paid or were given something in return?	1	No<br>Yes
bq104	How old were you the very first time you did any work or activity for pay or favours?	User entered integer	
bq105	In the last school term, have you done any chores or activities for which you got paid or were given something in return?		No<br>Yes
bq106_q	If yes, what were you given in return?	For display purpose only	
bq106_1	Personal clothes/things	0<br>1	No<br>Yes
bq106_2	School clothes	0<br>1	No<br>Yes
bq106_3	Food/drink	0<br>1	No<br>Yes
bq106_4	Money	0<br>1	No<br>Yes
bq106_5	Soap	0<br>1	No<br>Yes
bq106_6	Book	0<br>1	No<br>Yes
bq106_7	Phone	0<br>1	No<br>Yes
bq106_8	Pads for monthly period	0<br>1	No<br>Yes
bq106_9	Allowed to pass exams	0<br>1	No<br>Yes

Data Dictionary – Baseline Cups or Cash for Girls Trial sociodemographic and quality of life survey

Variable Name	Question Text	Saved Value	
bq106_10	House things	0	No
		1	Yes
bq106_11	Favours	0	No
		1	Yes
bq106_12	Less beatings	0	No
		1	Yes
bq106_13	Other	0	No
		1	Yes
bq107	If you do work, did you take time off school for this during the last school month?	0	No, not last month
			Yes, just a few times last month
		2	Yes, many times last month
		3	Yes, almost all the time last month
bq108	Have you ever had sex in order to pay for things, or get favours?	0	No
		1	Yes
bq109_q	If yes, what have you been given in return?	For display purpose only	
bq109_1	Personal clothes/things	0	No
		1	Yes
bq109_2	School clothes	0	No
		1	Yes
bq109_3	Food/drink	0	No
		1	Yes
bq109_4	Money	0	No
		1	Yes
bq109_5	Soap	0	No
		1	Yes
bq109_6	Book	0	No
		1	Yes
bq109_7	Phone	0	No
		1	Yes
bq109_8	Pads for monthly period	0	No
		1	Yes
bq109_9	Allowed to pass exams	0	No
		1	Yes
bq109_10	House things	0	No
		1	Yes

Data Dictionary – Baseline Cups or Cash for Girls Trial sociodemographic and quality of life survey

Variable Name	Question Text	Saved Value	
bq109_11	Favours	0<br>1	No<br>Yes
bq109_12	Less beatings	0<br>1	No<br>Yes
bq109_13	Other	0<br>1	No<br>Yes
bq116	Do you currently have any money saved?	0<br>1	No<br>Yes
bq118_q	Where do you usually get money†	For display purpose only	
bq118_1	Parents	0<br>1	No<br>Yes
bq118_2	Boyfriend/partner	0<br>1	No<br>Yes
bq118_3	Work	0<br>1	No<br>Yes
bq118_4	Other	0<br>1	No<br>Yes
bq118_oth	Please specify who else you get money from	User entered text	
bq120	If you get money from a man or boyfriend do they request sex in return?	0<br>1	No<br>Yes
ql1	It is hard for me to walk for more than 15 minutes	1<br>2<br>3<br>4<br>5	Never<br>Almost never<br>Sometimes<br>Almost always<br>Always
ql2	It is hard for me to run	1<br>2<br>3<br>4<br>5	Never<br>Almost never<br>Sometimes<br>Almost always<br>Always
ql3	It is hard for me to do sports activity or exercise	1<br>2<br>3<br>4<br>5	Never<br>Almost never<br>Sometimes<br>Almost always<br>Always
ql4	It is hard for me to lift something heavy	1<br>2<br>3<br>4<br>5	Never<br>Almost never<br>Sometimes<br>Almost always<br>Always

Data Dictionary – Baseline Cups or Cash for Girls Trial sociodemographic and quality of life survey

Variable Name	Question Text	Saved Value
qI5	It is hard for me to take a bath or shower by myself	1 Never<br>2 Almost never<br>3 Sometimes<br>4 Almost always<br>5 Always
qI6	It is hard for me to do chores around the house	1 Never<br>2 Almost never<br>3 Sometimes<br>4 Almost always<br>5 Always
qI7	I hurt or ache	1 Never<br>2 Almost never<br>3 Sometimes<br>4 Almost always<br>5 Always
qI8	I have low energy	1 Never<br>2 Almost never<br>3 Sometimes<br>4 Almost always<br>5 Always
qI9	I feel afraid or scared	1 Never<br>2 Almost never<br>3 Sometimes<br>4 Almost always<br>5 Always
qI10	I feel sad or blue	1 Never<br>2 Almost never<br>3 Sometimes<br>4 Almost always<br>5 Always
qIII	I feel angry	1 Never<br>2 Almost never<br>3 Sometimes<br>4 Almost always<br>5 Always
qI12	I have trouble sleeping	1 Never<br>2 Almost never<br>3 Sometimes<br>4 Almost always<br>5 Always
qI13	I worry about what will happen to me	1 Never<br>2 Almost never<br>3 Sometimes

Variable Name	Question Text	Saved Value	
		4	Almost always
		5	Always
qI14	I have trouble getting along with other teens	1	Never
		2	Almost never
		3	Sometimes
		4	Almost always
		5	Always
qI15	Other teens do not want to be my friend	1	Never
		2	Almost never
		3	Sometimes
		4	Almost always
		5	Always
qI16	Other teens tease me	1	Never
		2	Almost never
		3	Sometimes
		4	Almost always
		5	Always
qI17	I cannot do things that other teens my age can do	1	Never
		2	Almost never
		3	Sometimes
		4	Almost always
		5	Always
qI18	It is hard to keep up with my peers	1	Never
		2	Almost never
		3	Sometimes
		4	Almost always
		5	Always
qI19	It is hard to pay attention in class	1	Never
		2	Almost never
		3	Sometimes
		4	Almost always
		5	Always
qI20	I forget things	1	Never
		2	Almost never
		3	Sometimes
		4	Almost always
		5	Always
qI21	I have trouble keeping up with my school work	1	Never
		2	Almost never

Data Dictionary – Baseline Cups or Cash for Girls Trial sociodemographic and quality of life survey

Variable Name	Question Text	Saved Value	
		3	Sometimes
		4	Almost always
		5	Always
qI22	I miss school because of not feeling well	1	Never
		2	Almost never
		3	Sometimes
		4	Almost always
		5	Always
qI23	I miss school to go to the doctor or hospital	1	Never
		2	Almost never
		3	Sometimes
		4	Almost always
		5	Always
