# Supplementary figures and images for: Factors associated with the prevalence of HIV, HSV-2, pregnancy, and reported sexual activity among adolescent girls in rural western Kenya: A cross-sectional analysis of baseline data in a cluster randomized controlled trial
Source: PLoS Med. 2021 Sep 28;18(9):e1003756. doi: 10.1371/journal.pmed.1003756 (PMC8478198; doi:10.1371/journal.pmed.1003756)

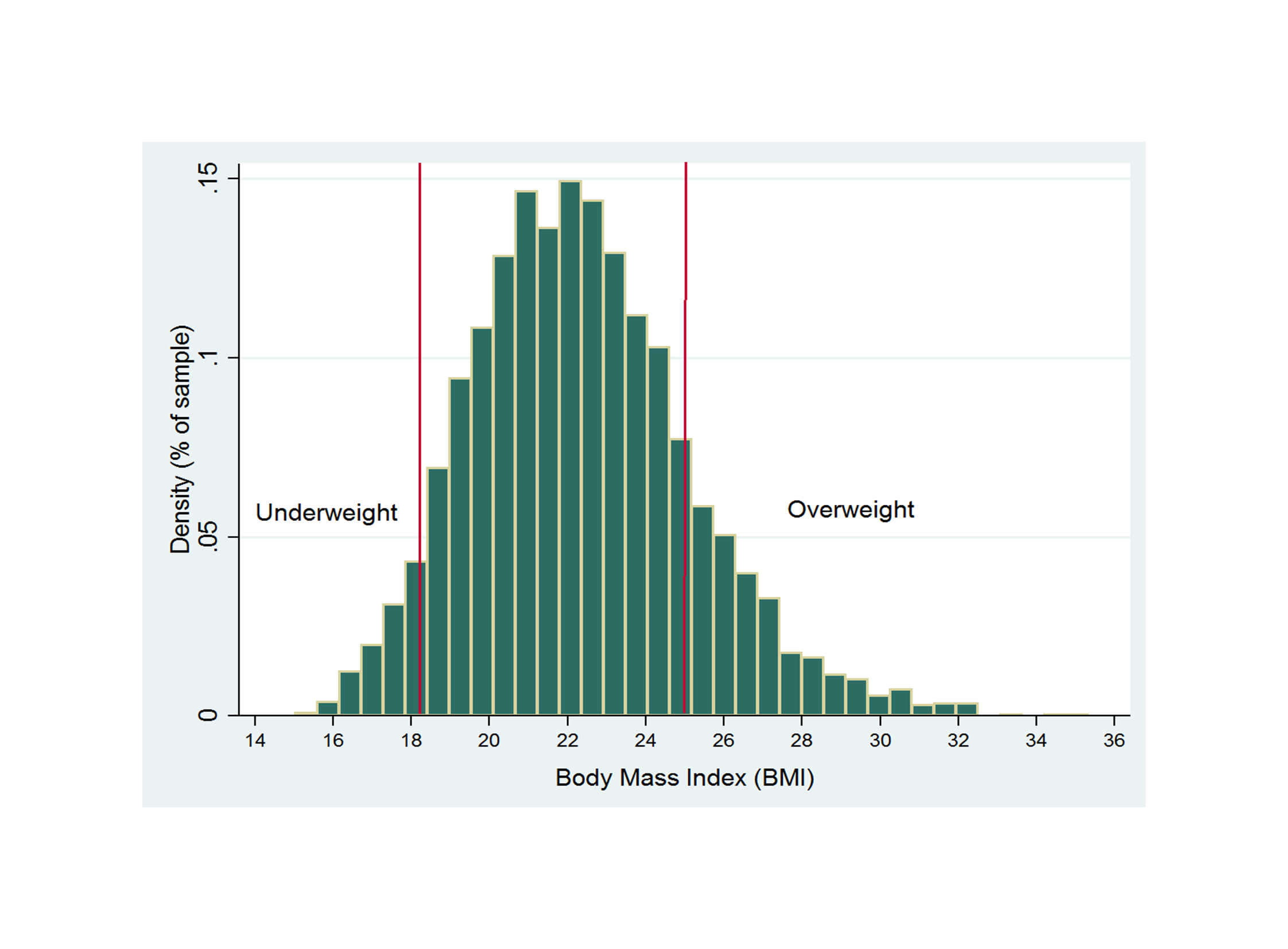

Supplement: S1 Fig — BMI classifications were based on percentile ranks: “underweight” classified as BMI <18.2, “normal weight” as BMI 18.2–25, and “overweight” as BMI >25. BMI, body mass index. (TIF) [file pmed.1003756.s002.tif]

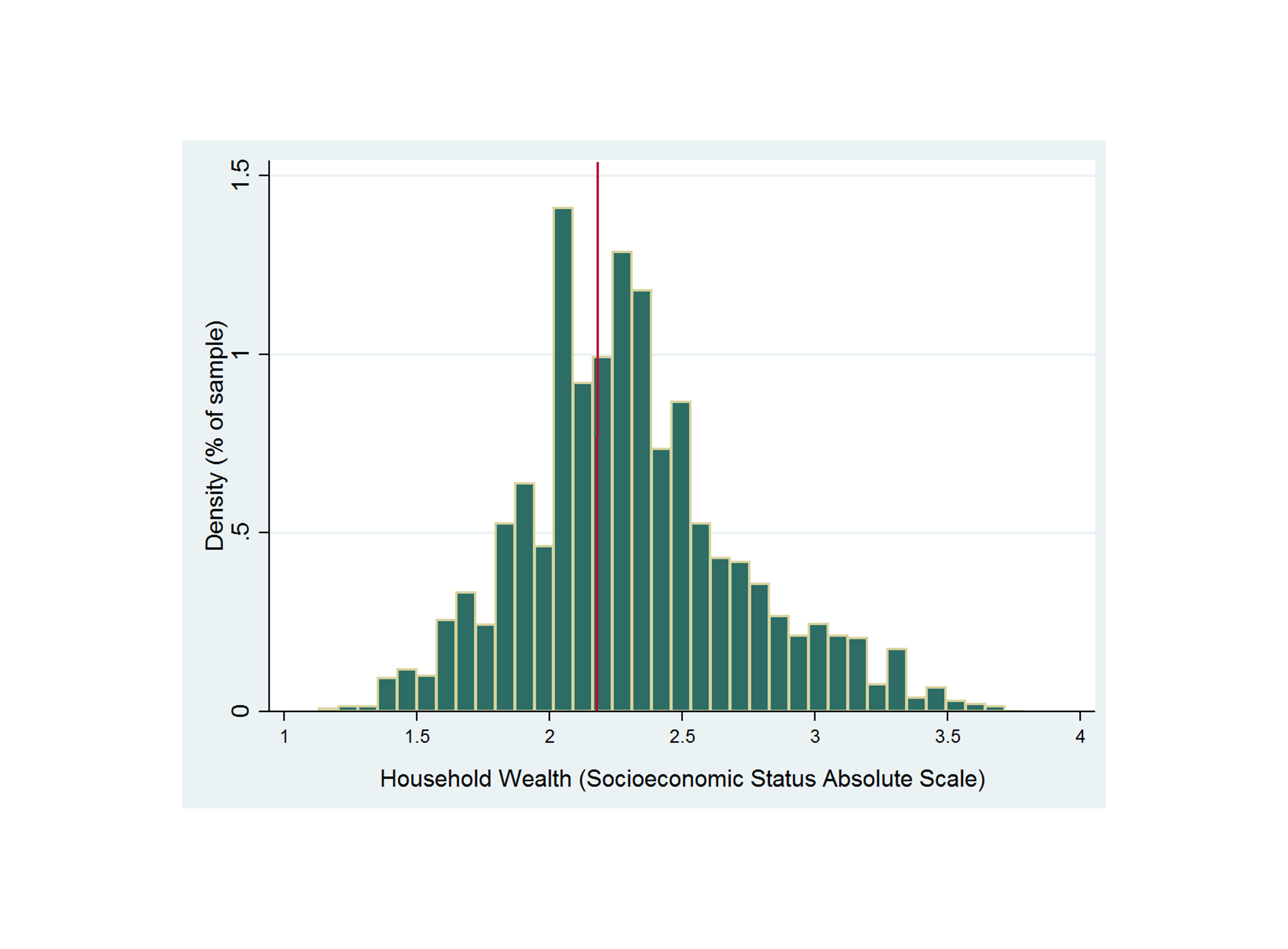

Supplement: S2 Fig — Absolute index based on girls reported household assets (Kabudula, 2017): Red reference line separates girls in households designated to the bottom 2 wealth quintiles from those living in households in the top 3 quintiles. (TIF) [file pmed.1003756.s003.tif]
