# Supplementary material for: Factors associated with the prevalence of HIV, HSV-2, pregnancy, and reported sexual activity among adolescent girls in rural western Kenya: A cross-sectional analysis of baseline data in a cluster randomized controlled trial
Source: PLoS Med. 2021 Sep 28;18(9):e1003756. doi: 10.1371/journal.pmed.1003756 (PMC8478198; doi:10.1371/journal.pmed.1003756)
Supplement: S1 Table — Bootstrapped replications using STATA swboot were performed to validate variable selection of stepwise models; bold indicates concordance with stepwise models, while (*) indicates a deviation from stepwise model. 0Overall well-being was measured through the 23-item Pediatric Quality of Life Inventory (PedsQL). (DOCX) [file pmed.1003756.s004.docx]

| **Variable Selection** | **Sexual Activity** | **Pregnancy** | **HIV** | **HSV-2** |
| --- | --- | --- | --- | --- |
|  | # of times selected | # of times selected | # of times selected | # of times selected |
| Age (year) | **1000** | **1000** | **963** | **854** |
| Early menarche (<13 years) / Age at menarche | 240 | **717** | 321 | **815** |
| Body Mass Index (BMI) | 560 | 105 | **785** | **809** |
| Marital status (MCW/SO) | 473 | 73 | 398 | 211 |
| Orphan (no living parent) | 100 | 107 | **714** | 211 |
| Drinking (self-report) | 190 | 0 | 0 | 2 |
| Socioeconomic status (SES) (poorest/ less poor) | 127 | 544 | 370 | 120 |
| Work (last seven days) | **750** | 106 | 178 | 347 |
| Received money from parents | 637* | 654 | 245 | 111 |
| Received money from boyfriend/partner | **1000** | 195 | 294 | **859** |
| Received money from working | **992** | 293 | 149 | 120 |
| Indecent touching | **1000** | **979** | 254 | 144 |
| Harassment for sex at school | **992** | **696** | 106 | 589 |
| Harassment for sex out of school | **1000** | 114 | 343 | 117 |
| Happy at home | 245 | **961** | 223 | 351 |
| Happy at school | 181 | 609 | 25 | 171 |
| Overall wellbeing⁰ | 255 | 138 | 66 | 170 |
| Engaged in sex for things or favours | -- | 427 | 95 | 361 |
| Did something to get pads | **873** | **728** | 310 | 126 |
| Used sanitary pads to manage entire period | 115 | 125 | 268 | 171 |
| Period stopped activities | 415 | 206 | **667** | 184 |
| Period severity | 508 | 146 | **554** | 180 |
| Period duration | 113 | 135 | **637** | 311 |
| Missed school during period (all reasons) | 407 | -- | 95 | 207 |
| Self-reported sex | -- | -- | 134 | 118 |
| Self-reported history of pregnancy | -- | -- | 119 | **910** |
| HIV seropositive | 123 | 89 | -- | 403 |
| HSV-2 seropositive | 170 | **796** | 299 | -- |

**S1 Table: Step-wise model variable selection check via ‘swboot’ bootstrap replications**

Footnote: Bootstrapped replications using STATA swboot were performed to validate variable selection of step-wise models; bold indicates concordance with step-wise models while (*) indicates a deviation from step-wise model. ⁰Overall wellbeing was measured through the 23-item Pediatric Quality of Life Inventory (PedsQL).
