# Supplementary material for: Factors associated with the prevalence of HIV, HSV-2, pregnancy, and reported sexual activity among adolescent girls in rural western Kenya: A cross-sectional analysis of baseline data in a cluster randomized controlled trial
Source: PLoS Med. 2021 Sep 28;18(9):e1003756. doi: 10.1371/journal.pmed.1003756 (PMC8478198; doi:10.1371/journal.pmed.1003756)
Supplement: S2 Table — (PDF) [file pmed.1003756.s005.pdf]

STROBE Statement—Checklist of items that should be included in reports of *cross-sectional studies*

	Item No	Recommendation
**Title and abstract**	1	(*a*) Indicate the study's design with a commonly used term in the title or the abstract
		(*b*) Provide in the abstract an informative and balanced summary of what was done and what was found
Background/rationale (Introduction paragraphs 1-4)	2	Explain the scientific background and rationale for the investigation being reported
Objectives (Abstract paragraph 1, Introduction paragraph 5)	3	State specific objectives, including any prespecified hypotheses
Study design (Methods paragraph 1)	4	Present key elements of study design early in the paper
Setting (Methods paragraph 2-4)	5	Describe the setting, locations, and relevant dates, including periods of recruitment, exposure, follow-up, and data collection
Participants (Methods paragraph 5-6)	6	(*a*) Give the eligibility criteria, and the sources and methods of selection of participants
Variables (Methods paragraph 10-11)	7	Clearly define all outcomes, exposures, predictors, potential confounders, and effect modifiers. Give diagnostic criteria, if applicable
Data sources/ measurement (Methods paragraph 7)	8*	For each variable of interest, give sources of data and details of methods of assessment (measurement). Describe comparability of assessment methods if there is more than one group
Bias (Methods paragraph 9)	9	Describe any efforts to address potential sources of bias
Study size (Methods paragraph 5)	10	Explain how the study size was arrived at
Quantitative variables (Methods paragraph 12-13)	11	Explain how quantitative variables were handled in the analyses. If applicable, describe which groupings were chosen and why
Statistical methods (Methods paragraph 14)	12	(*a*) Describe all statistical methods, including those used to control for confounding
		(*b*) Describe any methods used to examine subgroups and interactions
		(*c*) Explain how missing data were addressed
		(*d*) If applicable, describe analytical methods taking account of sampling strategy
		(*e*) Describe any sensitivity analyses
Participants (Methods Figure 1, Results paragraph 1)	13*	(a) Report numbers of individuals at each stage of study—eg numbers potentially eligible, examined for eligibility, confirmed eligible, included in the study, completing follow-up, and analysed
		(b) Give reasons for non-participation at each stage
		(c) Consider use of a flow diagram
Descriptive data (Results Table 1-2, Results paragraphs 1-2, 7)	14*	(a) Give characteristics of study participants (eg demographic, clinical, social) and information on exposures and potential confounders
		(b) Indicate number of participants with missing data for each variable of interest
Outcome data	15*	Report numbers of outcome events or summary measures

(Results Tables 3-5)		
Main results<br>(Results Tables 3-5,<br>Results paragraphs 3-6)	16	(*a*) Give unadjusted estimates and, if applicable, confounder-adjusted estimates and their precision (eg, 95% confidence interval). Make clear which confounders were adjusted for and why they were included
		(*b*) Report category boundaries when continuous variables were categorized
		(*c*) If relevant, consider translating estimates of relative risk into absolute risk for a meaningful time period
Other analyses<br>(Results paragraphs 8-9)	17	Report other analyses done—eg analyses of subgroups and interactions, and sensitivity analyses
Key results<br>(Discussion paragraph 1)	18	Summarise key results with reference to study objectives
Limitations<br>(Discussion paragraph 9)	19	Discuss limitations of the study, taking into account sources of potential bias or imprecision. Discuss both direction and magnitude of any potential bias
Interpretation<br>(Conclusion paragraph 1)	20	Give a cautious overall interpretation of results considering objectives, limitations, multiplicity of analyses, results from similar studies, and other relevant evidence
Generalisability<br>(Discussion paragraph 9)	21	Discuss the generalisability (external validity) of the study results
Funding<br>(**Funding information)	22	Give the source of funding and the role of the funders for the present study and, if applicable, for the original study on which the present article is based

***** details available in online portal and in published paper.***

Note: An Explanation and Elaboration article discusses each checklist item and gives methodological background and published examples of transparent reporting. The STROBE checklist is best used in conjunction with this article (freely available on the Web sites of PLoS Medicine at http://www.plosmedicine.org/, Annals of Internal Medicine at http://www.annals.org/, and Epidemiology at http://www.epidem.com/). Information on the STROBE Initiative is available at www.strobe-statement.org.
