# Supplementary material for: Factors associated with the prevalence of HIV, HSV-2, pregnancy, and reported sexual activity among adolescent girls in rural western Kenya: A cross-sectional analysis of baseline data in a cluster randomized controlled trial
Source: PLoS Med. 2021 Sep 28;18(9):e1003756. doi: 10.1371/journal.pmed.1003756 (PMC8478198; doi:10.1371/journal.pmed.1003756)
Supplement: S3 Table — CI, confidence interval; MCW, married, cohabitating, widowed; RR, risk ratio; SO, single, other. †Girls could list more than one source of money. 0Overall well-being was measured through the 23-item Pediatric Quality of Life Inventory (PedsQL). (DOCX) [file pmed.1003756.s006.docx]

| **Individual and Household**  **Characteristics** | **Sexual Activity** | | **Pregnancy** | | **HIV** | | **HSV-2** | |
| --- | --- | --- | --- | --- | --- | --- | --- | --- |
|  | **RR (95% CI)** | **P value** | **RR (95% CI)** | **P value** | **RR (95% CI)** | **P value** | **RR (95% CI)** | **P value** |
| Age (year) | 1.22 (1.19-1.26) | <0.001 | 1.54 (1.39-1.70) | <0.0001 | 1.28 (1.13-1.45) | <0.001 | 1.13 (1.08-1.18) | <0.001 |
| Age categorical |  |  |  |  |  |  |  |  |
| <16 | ref | ref | ref | ref | ref | ref | ref | ref |
| 16 | 1.41 (1.17-1.70) | <0.001 | 2.68 (0.76-9.45) | 0.126 | 0.67 (0.22-2.03) | 0.479 | 1.14 (0.90-1.44) | 0.282 |
| 17 | 1.88 (1.53-2.31) | <0.001 | 6.30 (1.94-20.45) | 0.002 | 1.58 (0.72-3.46) | 0.257 | 1.30 (1.03-1.63) | 0.026 |
| 18 | 2.45 (2.01-2.98) | <0.001 | 14.88 (4.74-46.71) | <0.001 | 2.20 (0.91-5.36) | 0.081 | 1.44 (1.16-1.79) | 0.001 |
| 19+ | 2.92 (2.41-3.53) | <0.001 | 38.75 (12.29-122.21) | <0.001 | 2.19 (0.77-6.23) | 0.140 | 1.69 (1.27-2.24) | <0.001 |
| HIV seropositivity | 1.17 (0.84-1.64) | 0.360 | 1.37 (0.45-4.16) | 0.573 | -- | -- | 1.42 (0.90-2.25) | 0.133 |
| HSV-2 seropositivity | 1.15 (1.02-1.29) | 0.023 | 2.23 (1.59-3.11) | <0.001 | 1.54 (0.85-2.80) | 0.151 | -- | -- |
| Prior pregnancy | -- | -- | -- | -- | 1.38 (0.45-4.30) | 0.574 | 1.90 (1.48-2.42) | <0.001 |
| Age at menarche (year) | 0.97 (0.93-1.02) | 0.268 | 0.96 (0.80-1.15) | 0.649 | 1.11 (0.90-1.37) | 0.334 | 1.11 (1.03-1.18) | 0.003 |
| Early menarche (<13yr) | 1.34 (1.12-1.61) | 0.002 | 2.25 (1.36-3.73) | 0.002 | 1.35 (0.59-3.10) | 0.480 | 0.81 (0.57-1.15) | 0.231 |
| Body Mass Index (BMI) |  |  |  |  |  |  |  |  |
| Underweight (BMI <18.2) | 0.71 (0.52-0.95) | 0.022 | 0.74 (0.31-1.76) | 0.493 | 3.63 (1.20-10.99) | 0.022 | 0.82 (0.59-1.15) | 0.251 |
| Normal (BMI 18.2-25) | ref | ref | ref | ref | 1.87 (0.85-4.10) | 0.120 | ref | ref |
| Overweight (BMI >25) | 1.22 (1.10-1.35) | <0.001 | 1.33 (0.84-2.09) | 0.226 | ref | ref | 1.24 (1.04-1.46) | 0.014 |
| Marital status: MCW/SO | 1.25 (1.03-1.52) | 0.024 | 1.03 (0.56-1.93) | 0.914 | 1.69 (0.81-3.54) | 0.163 | 1.17 (0.90-1.52) | 0.242 |
| Baby at home to care for | 3.08 (2.77-3.42) | <0.001 | 86.03 (55.29-133.85) | <0.001 | 1.88 (0.72-4.94) | 0.200 | 2.00 (1.60-2.50) | <0.001 |
| Orphan (no living parent) | 1.06 (0.82-1.38) | 0.652 | 1.35 (0.51-3.56) | 0.542 | 3.95 (1.74-8.95) | 0.001 | 1.08 (0.73-1.60) | <0.690 |
| Drinking (self-report) | 2.21 (1.54-3.18) | <0.001 | 1.00 (1.00-1.00) | . | 4.09 (0.65-25.64) | 0.133 | 0.39 (0.06-2.65) | 0.334 |
| Smoking (self-report) | 0.92 (0.26-3.27) | 0.893 | 1.00 (1.00-1.00) | . | 1.00 (1.00-1.00) | . | 1.00 (1.00-1.00) | . |
| Socioeconomic status (SES) categorical by quintile |  |  |  |  |  |  |  |  |
| Quintile 1 | 1.22 (1.04-1.44) | 0.017 | 2.28 (1.37-3.80) | 0.002 | 0.85 (0.38-1.91) | 0.690 | 1.05 (0.84-1.31) | 0.678 |
| Quintile 2 | 1.12 (0.97-1.31) | 0.130 | 1.71 (1.03-2.86) | 0.039 | 0.96 (0.45-2.06) | 0.924 | 0.99 (0.82-1.19) | 0.878 |
| Quintile 3 | 1.05 (0.89-1.25) | 0.552 | 1.38 (0.79-2.42) | 0.263 | 0.63 (0.30-1.32) | 0.222 | 0.97 (0.76-1.22) | 0.772 |
| Quintile 4 | 1.01 (0.86-1.20) | 0.870 | 1.17 (0.64-2.14) | 0.612 | 0.53 (0.25-1.12) | 0.097 | 1.00 (0.80-1.26) | 0.977 |
| Quintile 5 | ref | ref | ref | ref | ref | ref | ref | ref |
| SES (poorest/ less poor) | 1.16 (0.64-2.11) | 0.631 | 1.68 (1.19-2.38) | 0.003 | 1.26 (0.73-2.18) | 0.401 | 1.02 (0.90-1.17) | 0.717 |
| Work (last seven days) | 1.36 (1.21-1.54) | <0.001 | 1.59 (1.12-2.27) | 0.010 | 0.80 (0.43-1.48) | 0.471 | 1.12 (0.92-1.37) | 0.247 |
| School absence during prior month due to work | 1.07 (0.85-1.36) | 0.550 | 1.02 (0.42-2.45) | 0.971 | 1.00 (1.00-1.00) | . | 1.05 (0.67-1.64) | 0.847 |
| Have money saved | 0.86 (0.73-1.01) | 0.071 | 0.81 (0.49-1.36) | 0.429 | 0.64 (0.29-1.43) | 0.276 | 1.08 (0.87-1.33) | 0.486 |
| Received money from parents† | 0.58 (0.50-0.67) | <0.001 | 0.34 (0.23-0.50) | <0.001 | 0.90 (0.35-2.36) | 0.834 | 0.86 (0.68-1.10) | 0.229 |
| Received money from boyfriend/partner† | 3.33 (3.05-3.63) | <0.001 | 3.52 (2.20-5.65) | <0.001 | 1.88 (0.83-4.24) | 0.129 | 1.54 (1.22-1.95) | <0.001 |
| Received money from working† | 1.62 (1.45-1.82) | <0.001 | 2.37 (1.63-3.44) | <0.001 | 0.85 (0.40-1.78) | 0.661 | 1.05 (0.85-1.29) | 0.661 |
| Touched indecently | 3.67 (3.33-4.04) | <0.001 | 3.21 (2.19-4.70) | <0.001 | 1.58 (0.93-2.68) | 0.089 | 1.12 (0.91-1.38) | 0.275 |
| Harassment for sex at school | 2.16 (1.93-2.42) | <0.001 | 0.88 (0.51-1.51) | 0.634 | 0.88 (0.40-1.96) | 0.762 | 0.85 (0.68-1.06) | 0.138 |
| Harassment for sex out of school | 2.42 (2.14-2.74) | <0.001 | 1.21 (0.87-1.68) | 0.258 | 0.67 (0.40-1.10) | 0.111 | 0.93 (0.81-1.07) | 0.332 |
| Happy at home (no) | 1.67 (1.47-1.90) | <0.001 | 3.50 (2.26-5.42) | <0.001 | 1.31 (0.59-2.94) | 0.508 | 0.99 (0.76-1.29) | 0.927 |
| Happy at school (no) | 1.43 (1.16-1.77) | 0.001 | 1.06 (0.43-2.59) | 0.905 | 0.52 (0.07-3.88) | 0.527 | 1.08 (0.74-1.57) | 0.706 |
| Overall wellbeing⁰ |  |  |  |  |  |  |  |  |
| Low wellbeing | 2.30 (1.35-3.93) | 0.002 | 1.00 (1.00-1.00) | . | 1.00 (1.00-1.00) | . | 1.95 (0.96-3.96) | 0.065 |
| Moderate-low wellbeing | 1.98 (1.69-2.33) | <0.001 | 2.54 (1.43-4.51) | 0.002 | 0.73 (0.23-2.33) | 0.592 | 1.12 (0.87-1.46) | 0.382 |
| Moderate wellbeing | 1.50 (1.35-1.67) | <0.001 | 1.27 (0.89-1.80) | 0.190 | 1.11 (0.76-1.62) | 0.585 | 0.98 (0.84-1.14) | 0.786 |
| High wellbeing | ref | ref | ref | ref | ref | ref | ref | ref |
| Engaged in sex for things or favours | 1.89 (1.53-2.33) | <0.001 | 4.36 (2.31-8.23) | <0.001 | 1.85 (0.25-13.51) | 0.542 | 1.60 (0.99-2.60) | 0.054 |
| Used sanitary pads to manage entire period | 0.85 (0.77-0.94) | 0.001 | 0.73 (0.48-1.09) | 0.124 | 1.31 (0.77-2.22) | 0.319 | 0.92 (0.79-1.07) | 0.272 |
| Period stopped activities | 1.58 (1.44-1.75) | <0.001 | 1.35 (0.98-1.87) | 0.065 | 0.47 (0.24-0.91) | 0.026 | 1.03 (0.88-1.20) | 0.718 |
| Period severity |  |  |  |  |  |  |  |  |
| Heavy | 1.34 (1.19-1.51) | <0.001 | 1.56 (1.09-2.22) | 0.015 | 0.92 (0.51-1.68) | 0.793 | 1.16 (0.99-1.35) | 0.062 |
| Normal | ref | ref | ref | ref | ref | ref | ref | ref |
| Light | 0.91 (0.73-1.14) | 0.402 | 1.09 (0.55-2.19) | 0.798 | 2.60 (1.37-4.90) | 0.003 | 1.12 (0.88-1.42) | 0.359 |
| Period duration |  |  |  |  |  |  |  |  |
| <3 days | 0.97 (0.69-1.37) | 0.857 | 1.50 (0.67-3.32) | 0.321 | 2.71 (1.18-6.23) | 0.019 | 1.03 (0.71-1.51) | 0.865 |
| 3-5 days | ref | ref | ref | ref | ref | ref | ref | ref |
| >5 days | 1.19 (1.07-1.33) | 0.002 | 1.59 (1.09-2.34) | 0.017 | 0.72 (0.32-1.61) | 0.424 | 1.16 (0.97-1.38) | 0.109 |
| Missed school during period (all reasons) | 1.65 (1.46-1.86) | <0.001 | 1.57 (1.06-2.31) | 0.023 | 0.56 (0.24-1.32) | 0.187 | 0.93 (0.77-1.12) | 0.424 |
| Missed school due to period – most recent period | 1.32 (1.03-1.69) | 0.026 | 0.61 (0.30-1.26) | 0.183 | 1.16 (0.13-10.17) | 0.891 | 1.38 (0.77-2.48) | 0.283 |
| Subcounty |  |  |  |  |  |  |  |  |
| Gem | 1.18 (0.92-1.53) | 0.195 | 1.17 (0.54-2.51) | 0.688 | 1.03 (0.42-2.53) | 0.952 | 0.72 (0.52-1.00) | 0.053 |
| Rarieda | 1.37 (1.06-1.77) | 0.017 | 1.05 (0.50-2.19) | 0.896 | 1.40 (0.58-3.40) | 0.452 | 0.81 (0.56-1.18) | 0.272 |
| Siaya | 1.19 (0.89-1.59) | 0.229 | 0.88 (0.41-1.89) | 0.739 | 1.05 (0.40-2.72) | 0.925 | 0.91 (0.65-1.28) | 0.589 |
| Ugenya | 1.12 (0.86-1.47) | 0.407 | 1.02 (0.46-2.23) | 0.970 | 0.86 (0.33-2.20) | 0.749 | 0.94 (0.68-1.29) | 0.685 |
| Ugunja | ref | ref | ref | ref | ref | ref | ref | ref |
|  |  |  |  |  |  |  |  |  |

**S3 Table: Univariate associations between individual and partner risk factors and outcomes of interest among 3998 adolescent girls in secondary school, Siaya County, western Kenya 2017-2018**

Footnote: Abbreviations – RR: Risk ratio; CI: Confidence interval; MCW: Married, cohabitating, widowed; SO: Single, Other; †girls could list more than one source of money; ⁰Overall wellbeing was measured through the 23-item Paediatric Quality of Life Inventory (PedsQL).
