# Supplementary material for: Factors associated with the prevalence of HIV, HSV-2, pregnancy, and reported sexual activity among adolescent girls in rural western Kenya: A cross-sectional analysis of baseline data in a cluster randomized controlled trial
Source: PLoS Med. 2021 Sep 28;18(9):e1003756. doi: 10.1371/journal.pmed.1003756 (PMC8478198; doi:10.1371/journal.pmed.1003756)
Supplement: S4 Table — CI, confidence interval; RR, risk ratio. (DOCX) [file pmed.1003756.s007.docx]

| **Sexual and Partner Risk Characteristics** | **Pregnancy** | | **HIV** | | **HSV-2** | |
| --- | --- | --- | --- | --- | --- | --- |
|  | **RR (95% CI)** | **P value** | **RR (95% CI)** | **P value** | **RR (95% CI)** | **P value** |
| Age (year) | 1.35 (1.24-1.47) | <0.001 | 1.05 (0.86-1.28) | 0.620 | 1.16 (1.10-1.23) | <0.001 |
| Early menarche (<13yr) | 1.69 (1.07-2.69) | 0.025 | 1.29 (0.29-5.68) | 0.733 | 1.09 (0.66-1.80) | 0.742 |
| Body Mass Index (BMI) |  |  |  |  |  |  |
| Underweight (BMI <18.2) | 1.04 (0.46-2.37) | 0.930 | 1.23 (0.19-8.00) | 0.830 | 0.42 (0.14-1.24) | 0.116 |
| Normal (BMI 18.2-25) | ref | ref | ref | ref | ref | ref |
| Overweight (BMI >25) | 1.09 (0.69-1.70) | 0.719 | 0.69 (0.20-2.39) | 0.553 | 1.49 (1.15-1.92) | 0.003 |
| Household SES (poorest vs less poor) | 1.47 (1.06-2.05) | 0.020 | 1.56 (0.63-3.90) | 0.340 | 1.01 (0.80-1.29) | 0.907 |
| Age at sexual debut (year) | 1.18 (1.09-1.28) | <0.001 | 0.94 (0.76-1.16) | 0.571 | 1.05 (0.98-1.13) | 0.188 |
| Early sexual debut (<15) | 0.47 (0.26-0.83) | 0.009 | 1.23 (0.40-3.80) | 0.720 | 0.62 (0.40-0.96) | 0.031 |
| First sex – not forced | 2.43 (1.70-3.48) | <0.001 | 2.38 (0.93-6.10) | 0.069 | 1.23 (0.96-1.57) | 0.102 |
| First sex - undesired | 0.92 (0.64-1.33) | 0.673 | 1.29 (0.38-4.39) | 0.681 | 0.70 (0.56-0.89) | 0.003 |
| Touched indecently | 0.88 (0.60-1.30) | 0.524 | 1.58 (0.93-2.68) | 0.089 | 1.12 (0.91-1.38) | 0.275 |
| Harassed for sex at school | 0.40 (0.23-0.69) | 0.001 | 0.81 (0.27-2.46) | 0.712 | 0.79 (0.61-1.04) | 0.089 |
| Harassed for sex out of school | 0.49 (0.36-0.68) | <0.001 | 0.79 (0.31-1.97) | 0.607 | 0.93 (0.81-1.07) | 0.332 |
| Happy at home (no) | 2.10 (1.39-3.17) | <0.001 | 1.31 (0.40-4.29) | 0.655 | 1.12 (0.78-1.61) | 0.552 |
| Happy at school (no) | 0.74 (0.30-1.83) | 0.510 | -- | -- | 0.82 (0.38-1.77) | 0.619 |
| Self-reported history of pregnancy | -- | -- | 1.26 (0.37-4.37) | 0.713 | 1.81 (1.37-2.40) | <0.001 |
| HIV seropositive | 1.23 (0.42-3.61) | 0.708 | -- | -- | 1.42 (0.90-2.25) | 0.133 |
| HSV-2 seropositive | 1.95 (1.41-2.69) | <0.001 | 1.54 (0.85-2.80) | 0.151 | -- | -- |
| Number of partners (6 months) |  |  |  |  |  |  |
| None | ref | ref | ref | ref | ref | ref |
| One | 1.10 (0.74-1.63) | 0.646 | 2.61 (0.58-11.72) | 0.210 | 1.13 (0.83-1.54) | 0.437 |
| Two or more | 0.80 (0.44-1.46) | 0.467 | 3.82 (0.76-19.28) | 0.104 | 0.91 (0.60-1.37) | 0.653 |
| Don't know |  |  |  |  |  |  |
| Number of lifetime partners |  |  |  |  |  |  |
| One | ref | ref | ref | ref | ref | ref |
| Two or more | 1.23 (0.88-1.72) | 0.218 | 1.18 (0.50-2.77) | 0.705 | 1.07 (0.80-1.42) | 0.658 |
| Don't know | 0.56 (0.27-1.15) | 0.112 | 1.14 (0.33-3.94) | 0.842 | 0.94 (0.64-1.37) | 0.735 |
| Age discordancy of partner |  |  |  |  |  |  |
| Younger than you | 0.81 (0.12-5.45) | 0.829 | -- | -- | 0.95 (0.26-3.44) | 0.932 |
| About the same age | ref | ref | ref | ref | ref | ref |
| Older than you by <5 | 1.71 (1.15-2.56) | 0.009 | 1.17 (0.41-3.35) | 0.773 | 1.14 (0.80-1.60) | 0.472 |
| Older than you by 5-9 | 1.75 (1.12-2.75) | 0.04 | 0.67 (0.09-5.12) | 0.696 | 0.85 (0.48-1.53) | 0.591 |
| Older than you by 10+ | 0.95 (0.47-1.91) | 0.877 | 0.62 (0.08-4.94) | 0.648 | 0.87 (0.50-1.49) | 0.606 |
| Age discordancy (older) | 1.56 (1.14-2.14) | 0.006 | 0.97 (0.37-2.51) | 0.947 | 1.02 (0.77-1.35) | 0.889 |
| Was partner… |  |  |  |  |  |  |
| Someone you knew | 2.36 (1.39-4.03) | 0.002 | 2.63 (0.65-10.64) | 0.176 | 1.20 (0.89-1.63) | 0.226 |
| Someone you didn't know | ref | ref | ref | ref | ref | ref |
| If someone you knew… |  |  |  |  |  |  |
| Partner | 1.54 (0.96-2.48) | 0.073 | 1.24 (0.41-3.76) | 0.706 | 1.75 (1.17-2.62) | 0.007 |
| Relative/Other | ref | ref | ref | ref | ref | ref |
| Partner circumcised |  |  |  |  |  |  |
| Yes | ref | ref | ref | ref | ref | ref |
| No | 1.56 (0.95-2.57) | 0.079 | 1.61 (0.36-7.17) | 0.532 | 0.87 (0.53-1.44) | 0.589 |
| Don't know | 0.61 (0.43-0.88) | 0.008 | 0.88 (0.36-2.13) | 0.773 | 0.76 (0.59-0.97) | 0.026 |
| Condom use (last 6 months) | 1.01 (0.69-1.47) | 0.958 | 0.68 (0.25-1.84) | 0.449 | 0.93 (0.74-1.18) | 0.549 |
| Family planning (any method) | 4.15 (2.96-5.83) | <0.001 | 2.37 (0.62-9.02) | 0.205 | 1.81 (1.23-2.67) | 0.003 |
| Engaged in sex for things or favours | 2.28 (1.17-4.43) | 0.015 | 1.85 (0.25-13.51) | 0.542 | 1.82 (1.01-3.29) | 0.046 |
| Money for sex with partner | 1.09 (0.51-2.30) | 0.830 | 0.84 (0.15-4.66) | 0.842 | 0.84 (0.53-1.32) | 0.452 |
| Did something to get pads | 1.45 (0.98-2.14) | 0.060 | 0.50 (0.21-1.19) | 0.117 | 1.07 (0.91-1.26) | 0.414 |
| Sex in exchange for pads | 0.93 (0.38-2.29) | 0.882 | 2.64 (0.29-24.12) | 0.390 | 0.77 (0.34-1.74) | 0.528 |

**S4 Table: Univariate associations between sexual risk factors and outcomes of interest among 1090 sexually active adolescent girls attending secondary school, Siaya County, western Kenya 2017-2018**

Footnote: Abbreviations – RR: Risk ratio; CI: Confidence interval
